# Caloric restriction reverses left ventricular hypertrophy through the regulation of cardiac iron homeostasis in impaired leptin signaling mice

**DOI:** 10.1038/s41598-020-64201-2

**Published:** 2020-04-28

**Authors:** Hyeong Seok An, Jong Youl Lee, Eun Bee Choi, Eun Ae Jeong, Hyun Joo Shin, Kyung Eun Kim, Kyung-Ah Park, Zhen Jin, Jung Eun Lee, Jin Sin Koh, Woori Kwak, Won-Ho Kim, Gu Seob Roh

**Affiliations:** 1 Department of Anatomy and Convergence Medical Science, College of Medicine, Gyeongsang National University, Jinju Gyeongnam, Republic of Korea; 2 Bio Anti-aging Medical Research Center, Institute of Health Sciences, College of Medicine, Gyeongsang National University, Jinju Gyeongnam, Republic of Korea; 3 Department of Thoracic and Cardiovascular Surgery, College of Medicine, Gyeongsang National University, Jinju Gyeongnam, Republic of Korea; 4 Division of Cardiology, Department of Internal Medicine, College of Medicine, Gyeongsang National University, Jinju Gyeongnam, Republic of Korea; 5 C&K genomics, Songpa-gu, Seoul, Republic of Korea; 6 Division of Cardiovascular Diseases, Center for Biomedical Sciences, National Institute of Health, Cheongju, Chungbuk Republic of Korea

**Keywords:** Cardiac hypertrophy, Obesity

## Abstract

Leptin-deficient and leptin-resistant mice manifest obesity, insulin resistance, and left ventricular hypertrophy (LVH); however, LVH’s mechanisms are not fully understood. Cardiac iron dysregulation has been recently implicated in cardiomyopathy. Here we investigated the protective effects of caloric restriction on cardiac remodeling in impaired leptin signaling obese mice. RNA-seq analysis was performed to assess the differential gene expressions in the heart of wild-type and ob/ob mice. In particular, to investigate the roles of caloric restriction on iron homeostasis-related gene expressions, 10-week-old ob/ob and db/db mice were assigned to ad libitum or calorie-restricted diets for 12 weeks. Male ob/ob mice exhibited LVH, cardiac inflammation, and oxidative stress. Using RNA-seq analysis, we identified that an iron uptake-associated gene, transferrin receptor, was upregulated in obese ob/ob mice with LVH. Caloric restriction attenuated myocyte hypertrophy, cardiac inflammation, fibrosis, and oxidative stress in ob/ob and db/db mice. Furthermore, we found that caloric restriction reversed iron homeostasis-related lipocalin 2, divalent metal transporter 1, transferrin receptor, ferritin, ferroportin, and hepcidin expressions in the heart of ob/ob and db/db mice. These findings demonstrate that the cardioprotective effects of caloric restriction result from the cellular regulation of iron homeostasis, thereby decreasing oxidative stress, inflammation, and cardiac remodeling. We suggest that decreasing iron-mediated oxidative stress and inflammation offers new therapeutic approaches for obesity-induced cardiomyopathy.

## Introduction

Disruption of leptin signaling leads to obesity-induced cardiac remodeling^1,2^, and this progression of cardiac hypertrophy to heart failure is a major contributor to morbidity and mortality in obese patients^3^. Although the pathophysiology of cardiac remodeling in obesity is complex, leptin-deficient (ob/ob) and leptin-resistant (db/db) signaling serve an important role in obesity-associated left ventricular hypertrophy (LVH)^1,2,4^. The cardiomyopathy that stems from disruption of leptin signaling is due to several factors including insulin resistance, myocardial steatosis, inflammation, oxidative stress, and direct effects of deficiencies in leptin or leptin receptors^1,5,6^. However, the precise role of leptin on obesity-induced cardiomyopathy is not completely understood.

Cardiac iron homeostasis is important to cardiac function^7^. Inflammation and oxidative stress are two major pathophysiological processes that occur in heart with iron overload^8^ and lipocalin 2 (LCN2) has emerged as a particularly important iron-transporting protein in obesity- or diabetes-associated inflammatory conditions^9^. Indeed, LCN2 is currently used as a biomarker of cardiovascular disease in obesity^10^. In particular, cellular iron homeostasis is controlled by iron storage protein ferritin and iron uptake proteins such as LCN2, transferrin receptor (Tfrc), divalent metal transporter1 (DMT1)^11^. Iron export protein ferroportin (FPN) and its regulator, hepcidin, are central to cardiac iron homeostasis^12^. However, their roles in iron uptake, storage, and export are unclear in obese impaired leptin signaling mice with LVH.

In metabolic diseases, caloric restriction (CR) is a drug-free treatment that ameliorates some of the cardiac hypertrophy associated with sirtuin 1 and peroxisome proliferator-activated receptor gamma coativator-1α (PGC-1α) signaling in db/db mice^13^. Although CR provides documented cardioprotective effects by suppressing the oxidative stress induced by hypertension^14^, little is known about its effects on cardiac iron homeostasis in obesity-induced LVH.

In this study, we demonstrated the Tfrc upregulation in the hearts of ob/ob mice using RNA-seq analysis. We explored whether iron-regulating proteins are involved in cardiac hypertrophy, inflammation, and oxidative stress and whether CR confers benefits in leptin-deficient ob/ob and leptin-resistant db/db mice via regulation of iron homeostasis. Here, we report that CR reverses obesity-induced LVH through the regulation of iron uptake, storage, and export.

## Results

### Leptin-deficient ob/ob mice are characterized by cardiac hypertrophy induced by inflammation and oxidative stress

**Figure 1:**
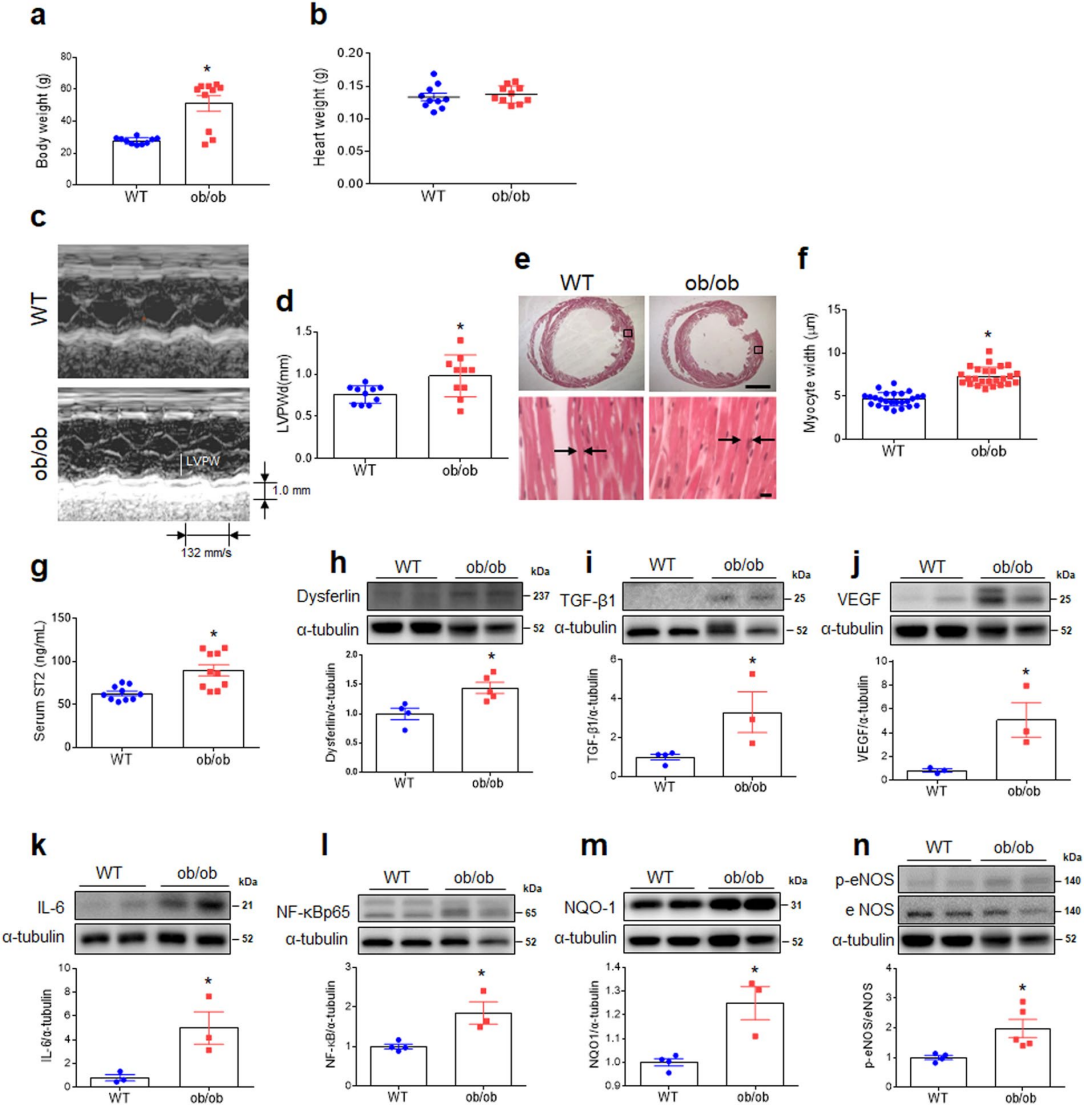
Effect of obesity on left ventricular hypertrophy and cardiac fibrosis in ob/ob mice. (**a**) Body weight, (**b**) Heart weight, (**c**) Representative images of echocardiography showing left ventricular wall, (**d**) Left ventricular posterior wall thickness at end-diastole (LVPWd) of heart as assessed by echocardiography, (**e**) Representative images of H&E staining in heart sections showing cardiomyocytes (scale bar = 1,000 μm [top], 10 μm [bottom]), (**f**) Cardiomyocyte width as measured from histological sections, (**g**) serum ST2 levels in wild-type (WT) and obese (ob/ob) mice (n = 10 per group). Western blots and quantitative analysis showing expression of cardiac fibrotic markers dysferlin (**h**), TGF-β1 (**i**), and VEGF (**j**) (band intensity normalized to α-tubulin; n = 3–5 mice per group). Western blots and quantitative analysis showing expression of cardiac inflammatory markers IL-6 (**k**) and NF-κBp65 (**l**). Western blots and quantitative analysis showing expression of cardiac oxidative stress markers NQO1 (**m**) and p-eNOS and eNOS (**n**) (n = 3–5 mice per group). Cropped blots are displayed here and full-length blots are included in the Supplementary Information. Data are shown as mean ± SEM. *P < 0.05 vs. WT. Statistical analysis was carried out by a Student’s t-test. Arrows denote borders of cardiomyocytes.

Although heart weight did not differ between wild type (WT) and ob/ob mice, the weights of body, liver, and epididymal fat pads in ob/ob mice were significantly higher than WT mice (Fig. 1a,b; Fig. S1). Echocardiography showed that ob/ob mice had significantly thicker posterior walls of the left ventricle (Fig. 1c,d), and histology indicated that cardiomyocyte width was also significantly greater in ob/ob than in WT mice (Fig. 1e,f). Serum soluble suppression of tumorigenicity 2 (ST2) levels were higher ob/ob than WT mice (Fig. 1g). Several biomarkers of fibrosis [dysferlin, transforming growth factor- β1 (TGF-β1), and vascular endothelial growth factor (VEGF)], inflammation [interleukin-6 (IL-6) and nuclear factor-kappaBp65 (NF-κBp65)], and oxidative stress [NAD(P)H dehydrogenase, quinone 1 (NQO1) and phosphorylated endothelial nitric oxide synthase (eNOS)] were greater in ob/ob than WT mice (Fig. 1h–n).

### Differential gene expression in the hearts of WT and ob/ob mice

**Figure 2:**
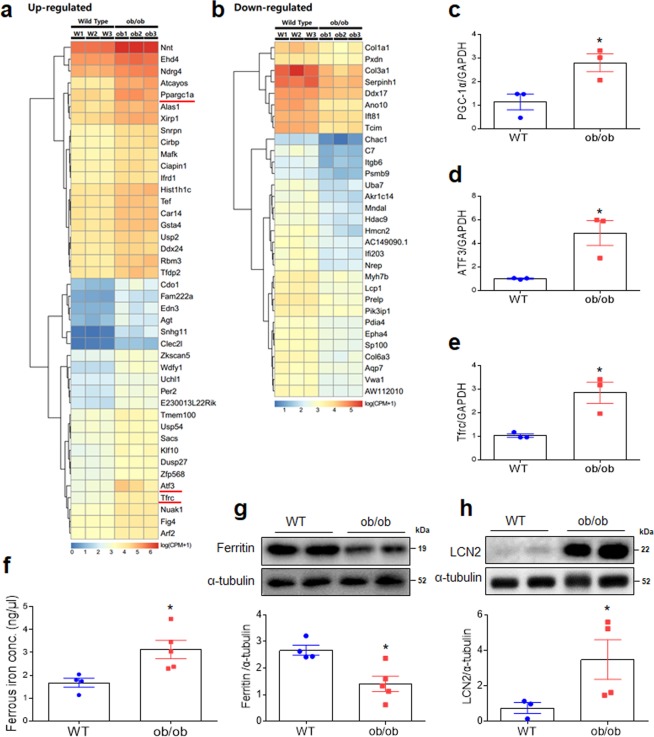
Effect of obesity on differential gene expression in the hearts of WT and ob/ob mice. Heatmap of differential gene expression showing genes (**a**) up- and (**b**) downregulated in ob/ob relative to WT mice (n = 3 mice per group). Genes were selected based on FDR value smaller than 0.001. Quantitative RT-PCR analysis of iron transport proteins PGC-1α (**c**), ATF3 (**d**), and Tfrc in the heart (**e**) (normalized to GAPDH; n = 3 mice per group). (**f**) Iron assay and quantitative analysis showing cardiac ferrous iron (n = 4–5 mice per group). Western blots and quantitative analysis showing expression of cardiac ferritin (**g**) and LCN2 (**h**) (n = 4–5 mice per group). Cropped blots are displayed here and full-length blots are included in the Supplementary Information. Data are shown as mean ± SEM. *P < 0.05 vs. WT. Statistical analysis was carried out by a Student’s t-test.

Numerous differences in gene expression related to leptin deficiency and obesity existed between WT and ob/ob mice (Fig. 2a,b; Fig. S2a–c; Tables S3 and S4). Among the many biological processes, cellular components, and molecular functions affected by this differential expression were genes with potential to cause cardiac hypertrophy, such as those involved in muscle structural development, mitogen-activated protein kinase phosphatase activity, and growth factor binding (Fig. S3). Notably, differences in expression levels among genes affecting inflammation and fibrosis were observed (Fig. 2a,b). A nearly threefold increase in transcription levels of PGC-1α and fivefold increase in activating transcription factor 3 (ATF3) was observed in ob/ob relative to WT mice, significant differences that were confirmed by qRT-PCR (Fig. 2c,d; Table S3). Furthermore, Tfrc mRNA was significantly greater in ob/ob than WT mice as assessed by RNA-seq and qRT-PCR (Fig. 2e; Table S3). An iron assay revealed a significant increase in cardiac ferrous iron in ob/ob mice relative to WT mice (Fig. 2f). Moreover, ferritin expression was significantly lower in the hearts of ob/ob than WT mice (Fig. 2g). LCN2 expression was significantly higher in ob/ob than WT mice (Fig. 2h).

### Effect of CR on cardiac hypertrophy by ameliorating fibrosis, inflammation, and oxidative stress in ob/ob and db/db mice

**Figure 3:**
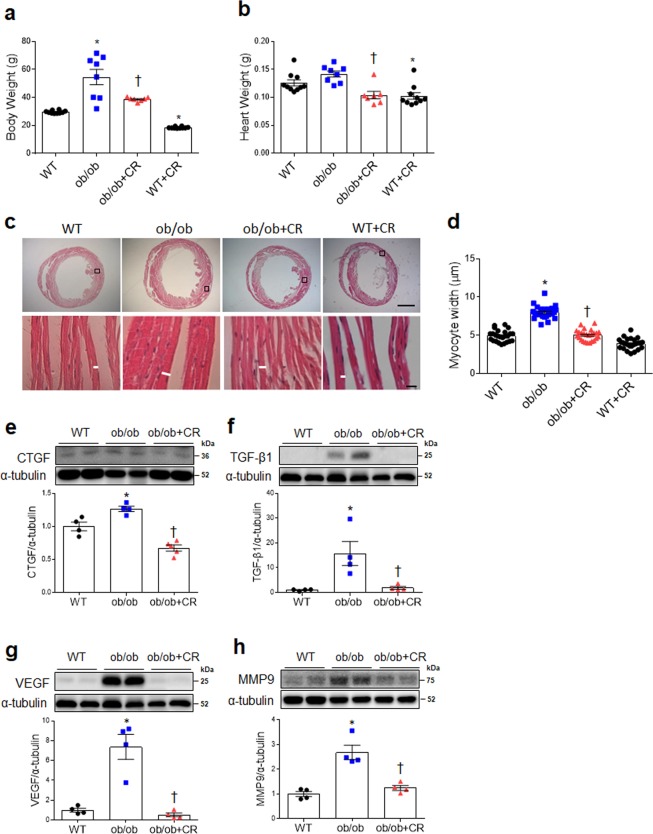
Effect of CR on cardiac hypertrophy and fibrosis in ob/ob mice. (**a**) Body weight and (**b**) heart weight in wild-type (WT), obese (ob/ob), obese calorie-restricted (ob/ob+CR), and calorie-restricted WT (WT + CR) mice (n = 8–10 per group). (**c**) Representative images of H&E staining in heart sections showing cardiomyocytes. White bar is cardiomyocyte width (scale bar = 1,000 μm [top], 10 μm [bottom]). (**d**) Cardiomyocyte width, as measured from histological sections. Western blots and quantitative analysis showing expression of cardiac fibrotic markers CTGF (**e**), TGF-β1 (**f**), VEGF (**g**), and MMP9 (**h**) (n = 4–5 per group). Cropped blots are displayed here and full-length blots are included in the Supplementary Information. Data are shown as mean ± SEM. *P < 0.05 vs. WT mice. ^†^P < 0.05 vs. ob/ob mice. Statistical analysis was carried out by one-way ANOVA followed by Bonferroni post hoc analysis.

Although there was significant increase of body weight in ob/ob and db/db mice relative to WT and db/m mice, there was no change of heart weight (Fig. 3a,b; Fig. S4a,b). However, CR significantly attenuated body and heart weights in ob/ob and db/db mice. In particular, cardiomyocyte diameter of CR mice was significantly decreased in both ob/ob and db/db lineages (Fig. 3c,d; Fig. S4c,d). Although CR reduced body and heart weights of WT and db/m mice, there was no reduction of cardiomyocyte diameter by CR in WT and db/m mice. Similarly, cardiac expression of fibrosis-related factors including connective tissue growth factor (CTGF), TGF-β1, VEGF, and matrix metalloproteinase9 (MMP9) were significantly downregulated after CR in ob/ob mice (Fig. 3e–h), reversing the patterns seen in ad libitum-fed counterparts. CR also significantly attenuated cardiac dysferlin and VEGF expression in db/db mice (Fig. S4e,f).

**Figure 4:**
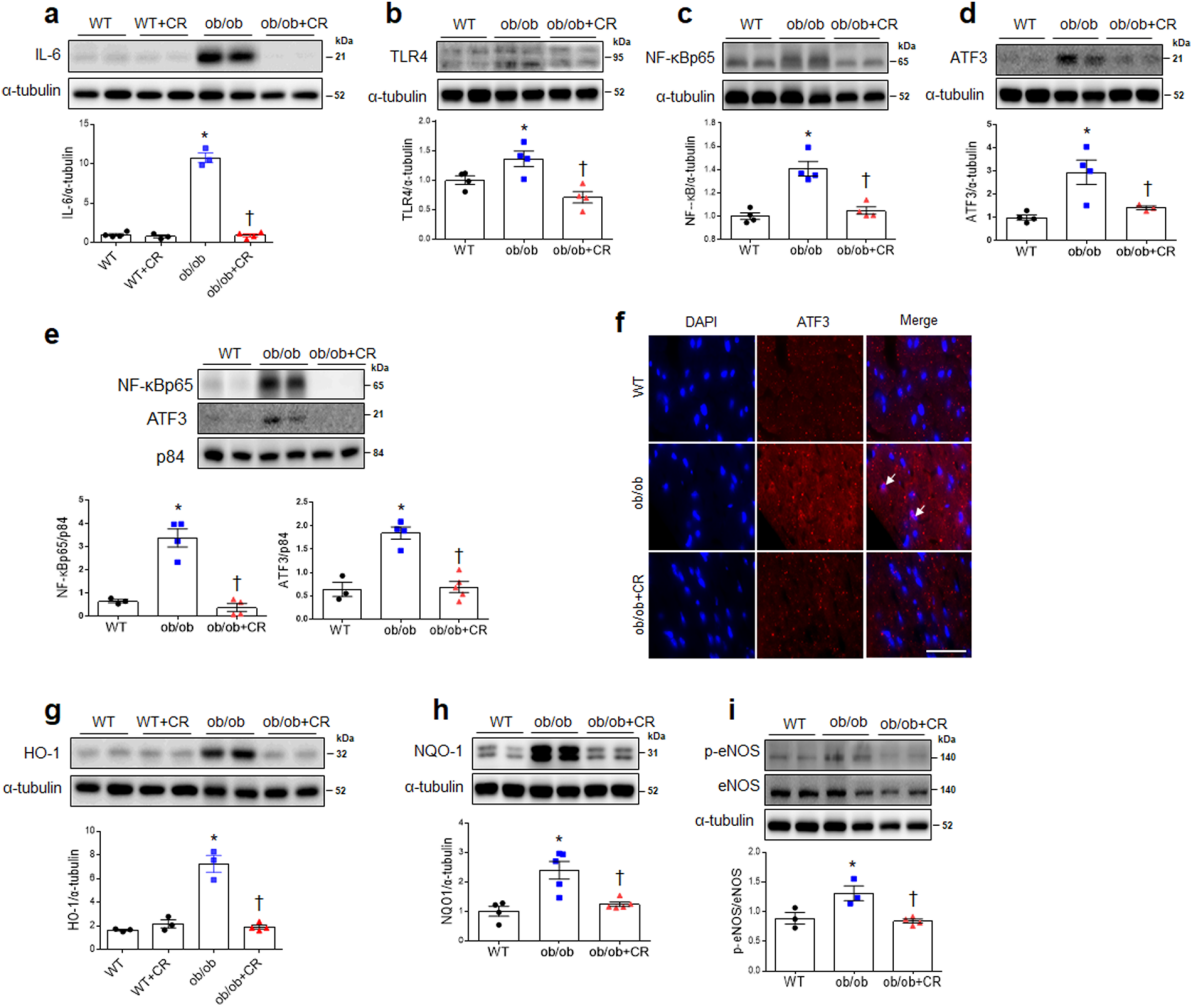
Effect of CR on cardiac inflammation and oxidative stress in ob/ob mice. Western blots and quantitative analysis showing expression of cardiac inflammation markers IL-6 (**a**), TLR4 (**b**), NF-κBp65 (**c**), and ATF3 (**d**) in wild-type (WT), obese (ob/ob), obese calorie-restricted (ob/ob+CR), and calorie– restricted WT (WT + CR) mice. (**e**) Western blots and quantitative analysis showing expression of cardiac nuclear NF-κBp65 and ATF3 (band intensity normalized to p84). (**f**) Representative images of immunofluorescence staining with ATF3 and DAPI from heart sections (scale bar = 25 μm; arrows: ATF3-positive cardiomyocytes). Western blots and quantitative analysis showing expression of cardiac antioxidants HO-1 (**g**), NQO-1 (**h**), and p-eNOS & eNOS (**i**). n = 3–5 per group for all assays. Cropped blots are displayed here and full-length blots are included in the Supplementary Information. Data are shown as mean ± SEM. *P < 0.05 vs. WT mice. ^†^P < 0.05 vs. ob/ob mice. Statistical analysis was carried out by one-way ANOVA followed by Bonferroni post hoc analysis.

Western blotting indicated that CR significantly decreased expression of IL-6 in ob/ob mice (Fig. 4a) and tended to decrease IL-6 expression in db/db mice, but this did not rise to the level of statistical significance (Fig, S5a). CR significantly attenuated upregulation of cardiac Toll like receptor-4 (TLR4) expression in the hearts of ob/ob mice (Fig. 4b), and NF-κBp65 expression was also significantly decreased in ob/ob and db/db mice after CR (Fig. 4c; Fig. S5b).

Total ATF3 expression levels were significantly higher in ob/ob mice compared to WT mice, but CR reversed its expression (Fig. 4d). Analysis of the nuclear fraction showed that ob/ob mice had high levels of ATF3 and NF-κBp65, but their expression levels were reversed by CR (Fig. 4e). Immunofluorescence staining showed that ATF3-positive cells were present in the hearts of ob/ob mice fed ad libitum but not in ob/ob+CR mice (Fig. 4f). CR also impacted the expression of anti-oxidant enzymes, as illustrated by western blot. In mice fed ad libitum, heme oxygenase-1 (HO-1) and NQO-1 levels were significantly higher in ob/ob and db/db relative to WT mice, but CR decreased their expression (Fig. 4g,h; Fig. S5c,d). CR also reversed the ratio of p-eNOS/eNOS in ob/ob mice (Fig. 4i). Furthermore, there was no change of IL-6 and HO-1 expressions in CR-treated WT and db/m mice (Fig. 4a,g, and Fig. S5a,c).

### Effect of CR on cardiac iron uptake in ob/ob and db/db mice

**Figure 5:**
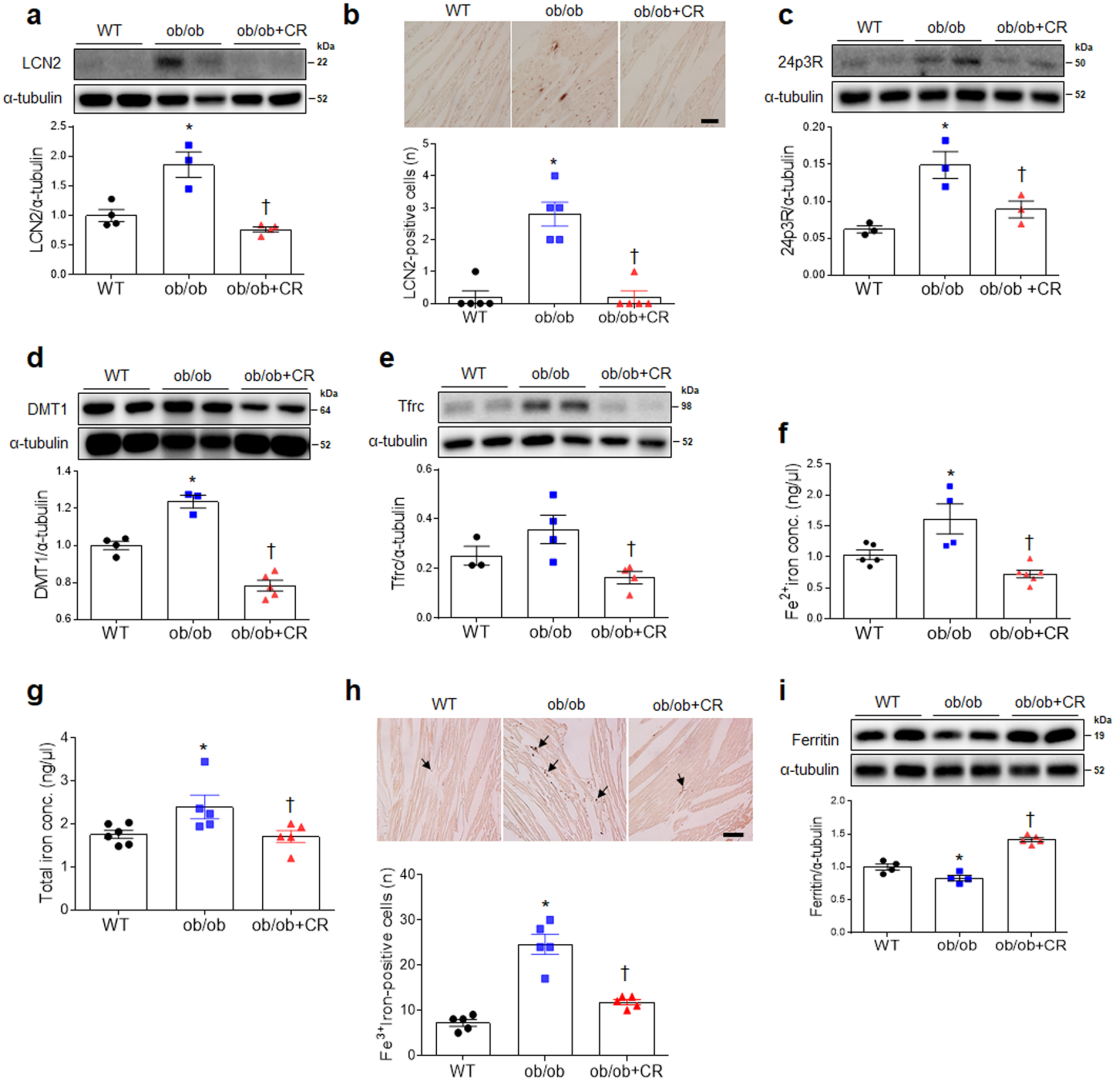
Effect of CR on iron transporter-related proteins in the hearts of ob/ob mice. (**a**) Western blots and quantitative analysis showing expression of cardiac LCN2 in wild-type (WT), obese (ob/ob), and obese calorie-restricted (ob/ob+CR) mice. (**b**) Representative images of immunohistochemistry for LCN2 in heart sections. The numbers of LCN2-positive cells were measured in LCN2-immunostained heart sections (Scale bar = 50 μm). Western blots and quantitative analysis showing expression of cardiac 24p3R (**c**), DMT1 (**d**), and Tfrc (**e**). Iron assay and quantitative analysis showing cardiac ferrous Fe^2+^ (**f**) and total iron (**g**) levels. (**h**) Representative images of DAB-enhanced Perls’ staining for ferric iron (Fe^3+^). The numbers of ferric iron-positvie cells were measured in Perls’ staining of hear sections. Arrows indicate iron deposits (scale bar = 50 μm). (**i**) Western blots and quantitative analysis showing cardiac ferritin expression in the hearts. n = 3–5 per group for all assays. Cropped blots are displayed here and full-length blots are included in the Supplementary Information. Data are shown as mean ± SEM. *P < 0.05 vs. WT mice. ^†^P < 0.05 vs. ob/ob mice. Statistical analysis was carried out by one-way ANOVA followed by Bonferroni post hoc analysis.

Due to our observation of elevated cardiac ferrous iron levels in ob/ob mice (Fig. 2f), we examined regulation of iron uptake-related genes via qRT-PCR. LCN2, DMT1, Tfrc, and L-ferritin mRNA levels were significantly lower in ob/ob+CR mice relative to ob/ob and db/db mice fed ad libitum (Figs. S6 and S8). Although the level of 24p3R mRNA in the hearts of ob/ob mice was significantly reduced by CR, we did not observe the same pattern in db/db mice (Figs. S6b and S8b). In addition, CR did not cause the change of iron uptake-related genes in WT and db/m mice (Figs. S6 and 8). As assessed by western blotting, CR reduced increased cardiac expression levels of LCN2 protein in ob/ob and db/db mice (Fig. 5a; Fig. S7a). Consistent with these findings, the numbers of immunostained LCN2-positive cells in heart sections of ob/ob mice were reduced by CR (Fig. 5b). Consistent with qRT-PCR findings (Figs. S6b and S8b), Western blotting indicated that high 24p3R expression levels were diminished in ob/ob+CR but not db/db+CR mice (Fig. 5c; Fig. S7b). CR inhibited DMT1 and Tfrc expression in the hearts of ob/ob and db/db mice (Fig. 5d,e; Fig. S7c,d), particularly decreasing cardiac ferrous iron (Fe^2+^) and total iron concentrations in ob/ob and db/db mice (Fig. 5f,g; Fig. S7e,f). As evident from DAB-enhanced Perls’ staining of the heart, CR reduced ferric iron (Fe^3+^) depositions in left ventricle sections of ob/ob and db/db mice (Fig. 5h; Fig. S7g). Furthermore, low expression of ferritin in ob/ob and db/db mice were significantly reversed by CR (Fig. 5i; Fig. S4e, S7h, and S8e). Tfrc and ferritin expressions are orchestrated in response to intracellular iron levels by two iron-regulatory proteins IRP1 and IRP2^15^. We found that that increased cardiac IRPs mRNA and protein levels in ob/ob and db/db mice were significantly attenuated by CR (Figs. S9 and 10). Consistent with these findings, the immunostained IRP1-positive cells in heart sections of ob/ob and db/db mice were reduced by CR (Figs. S9c and 10c).

### Effect of CR on cardiac iron export in ob/ob and db/db mice

**Figure 6:**
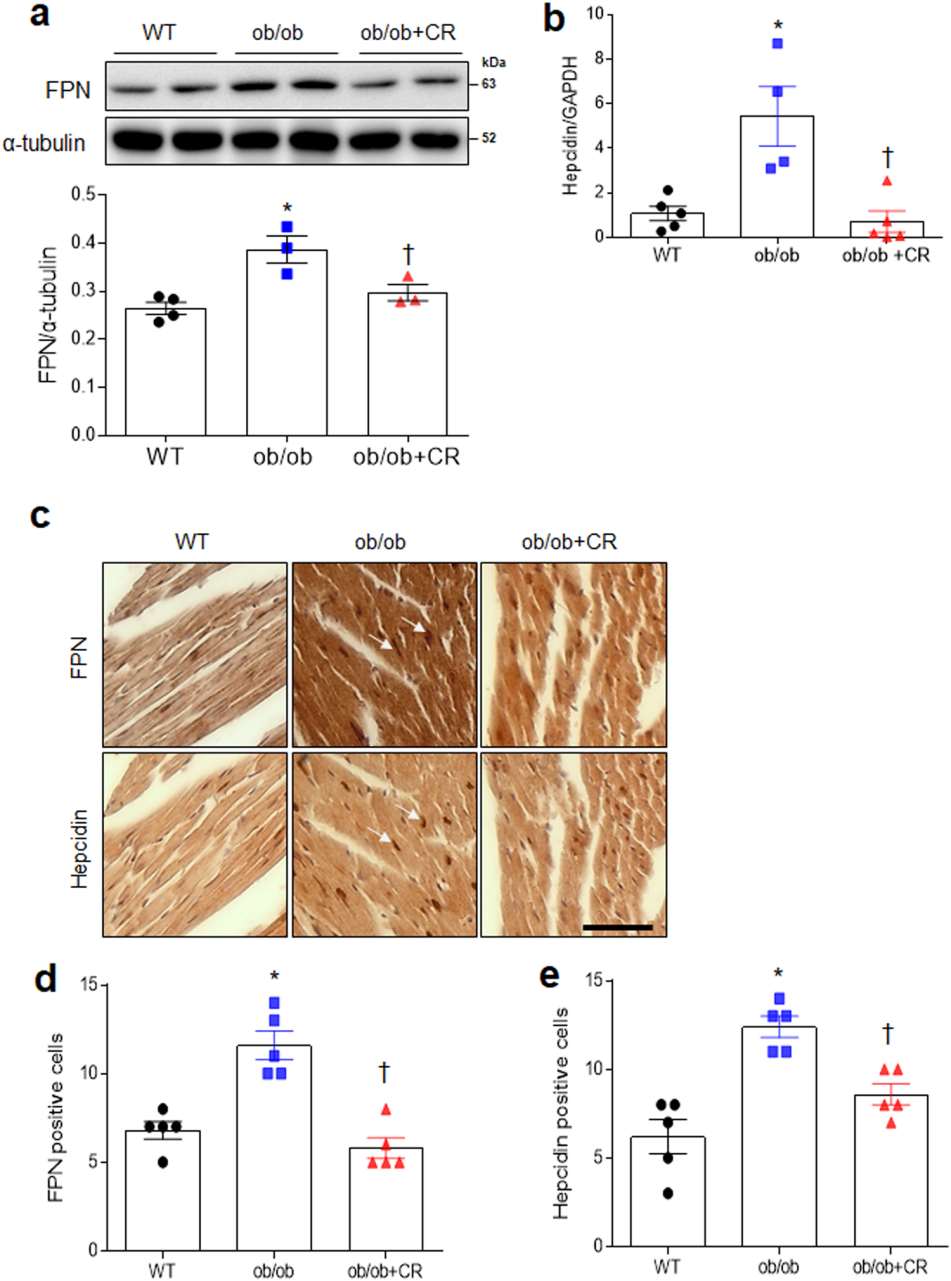
Effect of CR on cardiac ferroportin and hepcidin expressions in ob/ob mice. (**a**) Western blots and quantitative analysis showing cardiac ferroportin (FPN) expression in wild-type (WT), obese (ob/ob), and obese calorie-restricted (ob/ob+CR) mice. (**b**) Quantitative RT-PCR analysis of cardiac hepcidin. (**c**) Representative images of immunostained FPN and hepcidin from consecutive heart sections (Scale bar = 50 μm). The numbers of FPN (**d**)- and hepcidin (**e**)-positive cells were measured in FPN and hepcidin-immunostained heart sections. Arrows indicate the same FPN and hepcidin-positive cells in successive heart sections of ob/ob mice. Cropped blots are displayed here and full-length blots are included in the Supplementary Information. Data are shown as mean ± SEM. *P < 0.05 vs. WT mice. ^†^P < 0.05 vs. ob/ob mice. Statistical analysis was carried out by one-way ANOVA followed by Bonferroni post hoc analysis.

FPN is an iron-exporting transporter and is regulated by hepcidin, a hormone produced by the liver^16^. CR significantly decreased FPN expression in the hearts of ob/ob and db/db mice (Fig. 6a; Fig. S11a). In particular, increased hepcidin mRNA in ob/ob mice was attenuated by CR (Fig. 6b). Immunostained FPN and hepcidin-positive cells were observed in the consecutive heart sections (Fig. 6c). The numbers of immunostained FPN- or hepcidin-positive cells in the heart sections of ob/ob mice were reduced by CR (Fig. 6d,e). Immunohistochemistry also showed that hepcidin was more prevalent in the hearts of ad libitum-fed than db/db+CR mice (Fig. S11b). These findings indicate that CR could regulate cardiac iron concentrations by modulating expression of iron uptake, storage, and export proteins.

## Discussion

**Figure 7:**
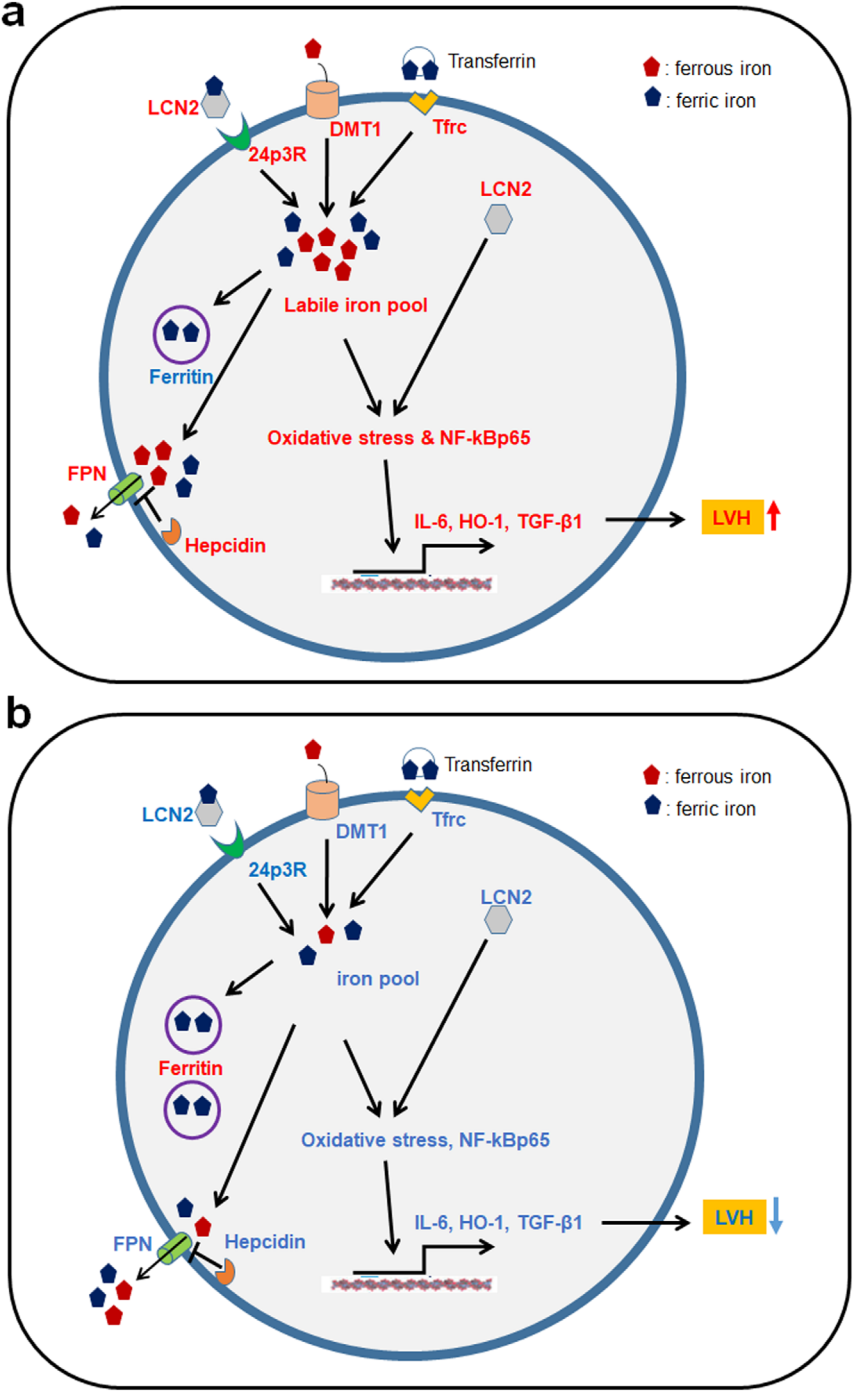
Schematic overview of effects of CR on cardiac iron homeostasis in ob/ob and db/db mice with left ventricular hypertrophy (LVH). (**a**) Obese LVH model compared with control mice. (**b**) Caloric restricted obese mice compared with obese mice. We propose a mechanism for LVH in which iron imbalance influences cardiomyocytes in ob/ob and db/db mice through elevated DMT1, Tfrc, and LCN2, which mediate the iron uptake in obesity. Furthermore, the balance of FPN and hepcidin determines a cardiac phenotype associated with iron overload because FPN is blocked by hepcidin. As ferritin expression decreases, ferrous iron is not stored as ferric iron, resulting in an increased labile iron pool and, ultimately, increased oxidative stress and NF-κBp65. Increased NF-κBp65 in turn upregulates expression of IL-6, HO-1, and VEGF, leading to LVH. However, CR reverses the obesity-induced cardiac iron overload, inflammation, and oxidative stress-related proteins in mice with disrupted leptin signaling. Increased proteins are shown in red and decreased in blue.

This study demonstrated that the cardiac hypertrophy, inflammation, and oxidative stress that characterize the hearts of ob/ob and db/db mice are reversed by CR. Furthermore, we demonstrated that cardiac iron imbalance of iron regulatory proteins results in cardiac iron overload-induced inflammation and oxidative stress, but these iron dynamics in the heart are reversibly controlled by CR. Specifically, we report that CR reverses aberrant cellular iron influx, storage, and export-associated proteins in obesity-induced LVH (Fig. 7). These findings have therapeutic potential given that CR can normalize iron overload in mice with disrupted leptin signaling to improve cardiac function.

Obesity is associated with cardiomyopathy and reduced longevity^17^. The majority of human obesity is due to leptin resistance rather than leptin deficiency, and the elevated plasma leptin that stem from these resistance correlates positively with cardiac hypertrophy^18^. However, leptin-deficient ob/ob mice also exhibit LVH, which can be reduced by treatment with leptin^2^, and similar morphological changes have been described in db/db mice^19^. On the other hand, two earlier studies reported that CR for 4 weeks does not significantly affect left ventricular mass in ob/ob mice^2,4^. The reasons for our different results are not clear, but may be due to three different factors including the amount of daily diet, duration of CR, and age-related changes of genetically obese mice. Taken together, these findings suggest that, regardless of leptin deficiency or resistance, obesity-induced other mediators will contribute to LVH.

In the present study, we found that 25-week-old ob/ob mice manifested NF-κBp65-mediated inflammation, oxidative stress, and LVH. These findings are consistent with evidence that progressive obesity develops in ob/ob and db/db mice at 6 months of age, but none of ob/ob mice have LVH at 2 months^2^. LVH is associated with fibrosis, which results in elevated dysferlin that functions in cardiac extracellular membrane repair, and TGF-β1 is increased in hyperglycemia in diabetic models^20,21^. Indeed, we found that soluble ST2, a fibrosis biomarker, is elevated in ob/ob mice at 25 weeks of age, which is consistent with findings reported from obese rats, in which serum ST2, collagen type 1, and profibrotic factors were elevated^22^. In stretch-induced cardiac hypertrophy, NF-κB activation is closely associated with VEGF secretion^23^, and these fibrogenic factors usually increase in wound healing after injury^24^. Here, a significant increase in ATF3 mRNA levels was observed using RNA-seq analysis. It has been supported that high-fat diet (HFD) causes increased ATF3 expression in cardiomyocytes and cardiac-specific ATF3 deletion increases severe cardiac fibrosis in HFD-fed mice^25^. Conversely, others have demonstrated that ATF3 deficiency reduces cardiac hypertrophy and protects the heart against pressure overload^26^. In this study, we found that CR inhibits increased cardiac ATF3 expression in ob/ob mice with LVH. Therefore, we demonstrate that CR may affect ATF3-mediated cardiac remodeling process in ob/ob mice.

In additional to its potential role as a regulator of fibrotic mediators, ATF3 is a target gene of NF-κB and is induced in when NF-κB is activated. In addition, ATF3 has been known as a negative regulator of cytokines such IL-6 and TNF-α in lipopolysaccharide-induced TLR4 signaling^27,28^. In this study, we demonstrated that CR attenuates the elevated cardiac IL-6 and NF-κBp65 expression in ob/ob and db/db mice. Indeed, we showed that cardiac NF-κBp65 expression increased in ob/ob mice, and that NF-κBp65 and ATF3 coexist in the nucleus of ob/ob mice, potentially due to the induction of ATF3 by oxidative stress or inflammation followed by localization to the nucleus to suppress NF-κBp65^29^. Therefore, our findings indicate that CR reverses NF-κBp65–mediated inflammation in obese-induced cardiac hypertrophy.

In addition to inflammation, obesity results in oxidative stress produced by excessive lipid peroxidation in mitochondria due to elevated glucose and lipid levels^30^. The accumulation of oxidative damage may contribute to cardiac inflammation and remodeling^31^ but the induction of antioxidants HO-1 and NQO1 exert anti-oxidative and anti-inflammatory functions^32^. HO-1 confers protection against cardiac injury, and its catalytic by-products (biliverdin and carbon monoxide) have anti-hypertrophic effects^33^. In the present study, we found that HO-1 and NQO1 were increased in ob/ob and db/db mice in response to oxidative stress, but CR attenuated these responses. These findings are supported by a previous study demonstrating that cilostazol attenuates doxorubicin-induced HO-1 and NQO-1 expression in cardiac fibrosis^20^ and that NQO1 and eNOS are activated in obesity in response to reactive oxygen species (ROS) to protect against oxidative stress^34^. Collectively, positioning our findings within existing research indicates that LVH is deeply associated with fibrosis, inflammation, and oxidative stress in obese mice with disrupted leptin signaling and that CR may be a potential anti-fibrotic, anti-inflammatory, and anti-oxidative treatment for cardiac remodeling.

Among the differential gene expressions using RNA-seq analysis, we found that iron-transporting Tfrc is significantly greater in the hearts of ob/ob mice compared to controls. Several iron transport mechanisms including Tfrc, DMT1, iron-siderophore-LCN2 complex, and iron exporter FPN involve in cardiomyocyte iron loading^35–38^. By reducing intracellular iron levels, cardiomyocytes protect from iron overload-induced oxidative stress. In patients with hereditary hemochromatosis and patients with β-thalassemia, iron overload exacerbated cardiac hypertrophy^8,39^. Intracellular ferrous iron—also called labile iron—is readily oxidized by the Haber-Weiss reaction, resulting in cellular ROS formation^40^; in the hearts of older rats, ferritin levels are increased, possibly as a protective response to oxidative stress from ROS accumulation^36,41^. In atherogenesis, increased ferritin expression following HO-1 induction is a major factor in protection against complement activation^42^. Thus, our data indicate that decreased ferritin expression can cause severe damage to cardiomyocytes in the heart of ob/ob and db/db mice.

Indeed, cardiac FPN expression is essential to intracellular iron homeostasis and to normal cardiac function^7^. Cardiac-specific deletion of FPN leads to fatal cardiac iron overload. However, we demonstrate that cardiac FPN expression is regulated in response to iron overload via iron uptake proteins including DMT1, Tfrc, and LCN2. Hepcidin, which inhibits the activity of FPN^12^, is involved in inflammation and its expression is increased by signaling transducer and activator of transcription3 (STAT3) signaling through cytokines such as IL-6^43^. The increase in hepcidin we observed is consistent with previous studies in models with chronic inflammation such as obese mice^44^. This finding is supported by previous research indicating that in morbidity obese women with non-alcoholic fatty liver disease, the hepatic mRNA expressions of hepcidin and FPN were significantly greater than in obese women with normal liver^45^. Although FPN inactivation by hepcidin results in displacement from the cellular membrane into the cytosol^46^, the activity of FPN may not coincide with the amount of FPN. Our findings indicate that there may be increased levels of inhibitory hepcidin expression in ob/ob mice as a compensatory reaction against increasing levels of cardiac FPN. Taken together, these findings indicate that disruptions to cardiac iron homeostasis via iron imbalance of iron uptake and export proteins result in cardiac iron overload-induced inflammation and oxidative stress but these iron dynamics in the heart are reversibly regulated by CR.

On the other hand, LCN2 is a well-documented component of the innate immune system that inhibits the growth of pathogenic bacteria by binding iron, and it has recently been implicated in iron homeostasis within hosts^47^. Levels of LCN2 in serum, liver, brain, and adipose tissue are elevated in several murine models of obesity^9,48–50^. In previous studies, we demonstrated that CR attenuates serum, liver, and hippocampus LCN2 levels in db/db and ob/ob mice^48,49^. As circulating LCN2 increases in patients with heart failure, the production of LCN2 in the heart increases^51^, and *in vitro* treatment of cardiomyocytes with LCN2 causes iron accumulation and oxidative stress through increased 24p3R expression^52,53^. LCN2 may also be related to LVH and heart failure^54^, serving as a risk marker for the progression of atherosclerosis due to LCN2-MMP9 complex formation^55^. Our findings show that elevated LCN2 and MMP9 expression in ob/ob mice was significantly reduced by CR. Thus, we suggest that LCN2 may be an emerging target for elucidating iron homeostasis and inflammation in obese mice with LVH.

In conclusion, multiple mechanisms result in cardiomyopathy in obesity including fibrosis, inflammation, and oxidative stress (Fig. 7). These pathologies can be duplicated in the hearts of ob/ob and db/db mice not only through disruption of leptin signaling, but also through interference with cardiac iron homeostasis via dysregulated iron uptake, storage, and export. However, CR prevents cardiac remodeling by preventing cardiac inflammation, fibrosis, and oxidative stress, and might be useful for the treatment of LVH, intervening in various pathways involved in the progression of obesity-induced cardiac remodeling.

## Materials and Methods

### Animals, caloric restriction, and study design

Male WT mice, ob/ob, db/m, and db/db mice from the C57BL/6 J background were purchased from Central Laboratory Animal Inc. (Seoul, South Korea) at 5 weeks of age and maintained in the animal facility at Gyeongsang National University (GNU). Animal experiments were performed in accordance with the National Institutes of Health Guide for the Care and Use of Laboratory Animals. The University Animal Care Committee for Animal Research of GNU approved the study protocol (GNU-150116-M0002). Mice were housed on a 12 h light/12 h dark cycle. In Experiment 1, 5 weeks old male WT and ob/ob mice were raised on a normal diet for 20 weeks for RNA-seq analysis (n = 10 mice per group). In Experiment 2, WT and ob/ob mice were fed ad libitum until 10 weeks of age, when they were randomly divided into four groups (n = 10 mice per group). Mice in both the WT and ob/ob groups continued to receive free access to food, but calorie-restricted ob/ob (ob/ob+CR) and WT (WT + CR) mice were transferred to individual cages and received a restricted amount of food (2 g food/day) for 12 weeks as previously described^48^. Although alternate feeding in rodents is as effective as CR in reducing obesity-induced body weight and glucose levels, extreme hunger while fasting indicate that this may not be a feasible regimen in orexigenic ob/ob mice^56^. This feeding restricted amounts in ob/ob mice maintained the body weight of ob/ob mice and further extended almost 50% of longevity comparable to that of mice fed ad libitum^57^. In Experiment 3, db/m and db/db mice were also randomly divided into four groups at 10 weeks of age; db/m and db/db mice were given free access to food and db/db+CR and db/m+CR mice were transferred to individual cages with restricted food (2 g/day) for 12 weeks, as previously described^49^.

### Transthoracic echocardiography

Heart functions of WT and ob/ob mice (n = 10 mice per group) were evaluated by transthoracic echocardiography at 25 weeks. Before echocardiography, mice were anesthetized with tiletamine hydrochloride/zolazepam hydrochloride (Zoletil, 0.25 mg/kg; Virbac Laboratories, Carros, France) by intramuscular injection. Two-dimensional and M-mode (one-dimensional) echocardiograms were assessed using a 12-MHz linear array transducer with a VIVID Q system (GE Healthcare, Wauwatosa, WI, USA). The left ventricle posterior wall thickness at end-diastole (LVPWd) were measured in the M-mode.

### Histological analysis

After an intraperitoneal injection of Zoletil (0.5 mg/kg; Virbac Laboratories, Carros, France), mice (n = 4 mice per group) were perfused with 4% paraformaldehyde in ice-cold 0.1 M phosphate-buffered saline (PBS) for tissue analysis. The hearts were fixed in 4% paraformaldehyde for 12 h at 4 °C. The samples were embedded in paraffin and cut into 5-µm sections. Section slides were then deparaffinized and stained with hematoxylin and eosin (H&E). The sections were visualized under a light microscope (BX51 Olympus, Tokyo, Japan), and digital images were captured and documented. Cardiomyocyte width was measured in longitudinal sections in myocardial regions with parallel myocyte fascicles. Five 200 × 200 μm^2^ fields were randomly selected on each section of LV wall (n = 4 mice per group) for evaluation. For detection of ferric iron, diaminobenzidine (DAB, Vector Laboratories, Burlingame, CA, USA)-enhanced Perls’ iron stain (Iron Stain Kit, ab150674, Abcam, Cambridge, MA, USA) was performed. Briefly, heart sections were incubated in Perls’ solution (Abcam) for 30 min, followed by incubation in 0.05% DAB (Vector Laboratories) for 20 min. Then, 1% hydrogen peroxide was added, and samples were incubated for 30 min. Sections were then washed with mounting medium and were visualized under a microscope (BX51, Olympus). DAB-enhanced Perls’ iron-positive cells were manually countered in LV region (200 × 200 μm^2^) in five sections using 40x objectives.

### Enzyme-linked immunosorbent assay (ELISA)

Cardiac hypertrophy is a type of cardiac remodeling driven by various fibrosis factors^58^. Concentration of serum ST2, a fibrosis biomarker, was measured using mouse ST2 (R&D Systems, MN, USA) ELISA kits according to the manufacturers’ protocols in 25-week-old WT and ob/ob mice (n = 10 mice per group).

### Iron assay

Cardiac ferrous and total iron levels from frozen heart were determined using the Iron Assay kit (MAK025, Sigma-Aldrich, St. Louis, MO, USA) according to the manufacturer’s protocol.

### Next generation sequencing (NGS)-based RNA-seq analysis

C&K Genomics (Seoul, South Korea) performed RNA-seq analysis from the hearts of WT and ob/ob mice at 25 weeks of age (n = 3 mice per group). The sequencing library was constructed using Illumina’s TruSeq RNA Prep kit, and data generation was performed following the manufacturer’s protocol with NextSeq. 500 platform. Raw data generated by this study is available in the NCBI database (Project Accession: PRJNA553625). The quality of generated data was verified using FastQC and Trimmomatic (v0.36), removing low-quality bases and adaptor contamination, and quality-filtered reads were mapped to the Mus musculus genome (GRCm38) from the Ensembl database using Hisat2 (Johns Hopkins University, v2.0.5). Mapped reads of expression for each gene were counted using Feature Count in R (R Foundation, v1.5.0) and EdgeR and DESeq. 2 were used for statistical analysis to identify genes expressed differentially between the two groups; the false discovery rate (FDR) was set to < 0.001. To identify the related functions of differentially expressed genes, an enrichment analysis of Gene Ontology (biological process, cellular component, and molecular function) was conducted using the Clue GO add-on module included in Cytoscape (Cytoscape Consortium, v3.7.1.).

### Quantitative reverse-transcription PCR (qRT-PCR)

Total mRNA from hearts was isolated using TRIzol (Invitrogen, Carlsbad, CA, USA) and reverse-transcribed using the RevertAid First-Strand cDNA Synthesis Kit (Fermentas, Inc., Hanover, MD, USA). Real-time RT-PCR was performed using the ABI Prism 7000 Sequence Detection System (Applied Biosystems, Foster City, CA, USA). PCR amplifications were performed using the SYBR Green I qPCR kit (TaKaRa, Shiga, Japan) with specific primers (Table S1) that targeted genes related to inflammation, oxidative stress, and iron transport, such as PGC-1α, associated with mitochondria biosynthesis and oxidative stresses^59^, activating transcription factor3 (ATF3), associated with stress response and glucose tolerance^25^, and Tfrc, an iron ion transporter^35^.

### Western blot analysis

For protein isolation, frozen heart tissues were homogenized in T-PER lysis buffer (25 mM bicine, 150 mM sodium chloride; pH 7.6, Pierce, Rockford, IL, USA). Homogenized tissues were incubated on ice for 20 min and sonicated three times. Samples were centrifuged for 30 min at 12,000 rpm at 4 °C, and supernatants were transferred to clean vials. For cytosolic and nuclear fraction preparations, trimmed hearts were lysed using ice-cold NE-PER buffer kit (Pierce) according to the manufacturers’ protocols. Samples were probed with primary antibodies (Table S2) for factors known to be associated with fibrosis and cardiac remodeling, such as dysferlin, CTGF, TGF-β1, and VEGF, and those associated with inflammation and oxidative stress, such as IL-6, NF-κBp65, TLR4, HO-1, NQO1, and eNOS. The membranes were visualized using an enhanced chemiluminescence substrate (Pierce). The Multi-Gauge image analysis program (Fujifilm, Tokyo, Japan, v3.0) was used to measure band densitometry. p84 or α-tubulin was used as an internal control to normalize protein levels.

### Immunofluorescence staining

Deparaffinized sections of heart were incubated with primary antibodies (Table S2) for double immunostaining. After washing three times with 0.1 M PBS, Alexa Fluor 488- and 594-conjugated donkey anti-rabbit or -mouse secondary antibodies (Invitrogen Life Technologies) were applied for 1 h at room temperature. Nuclei were stained with DAPI (1:10,000; Invitrogen). Fluorescence was visualized with a BX51-DSU microscope (Olympus), and digital images were captured.

### Immunohistochemical staining

Deparaffinized sections of heart were placed in 0.3% H_2_O_2_ for 20 min, washed, and incubated in blocking serum for 1 h at room temperature. Sections were incubated in primary antibodies (Table S2) at 4 °C overnight and with a secondary biotinylated antibody for 1 h at room temperature. After washing, sections were incubated in an avidin-biotin-peroxidase complex solution (Vector Laboratories,) and developed with 0.05% DAB substrate kit (Vector Laboratories). The sections were then dehydrated in graded alcohols, cleared in xylene, and mounted under a coverslip with Permount (Sigma-Aldrich). Sections were visualized under a BX51 light microscope (Olympus). Immunostained LCN2-, FPN-, and hepcidin-positive cells were manually countered in LV region (200 × 200 μm^2^) in five sections using 40x objectives.

### Statistical analysis

Statistical analyses were performed using PRISM (GraphPad Software Inc., San Diego, CA, USA). Group differences were determined by Student’s t-tests followed by unpaired-test analysis in WT and ob/ob mice and were determined by one-way ANOVA followed by Bonferroni post hoc analysis in ob/ob+CR and db/db+CR mice. All values are expressed as mean ± SEM. A P-value of less than 0.05 was considered statistically significant.

### Ethics approval and consent to participate

Animal experiments were performed in accordance with the National Institutes of Health Guide for the Care and Use of Laboratory Animals. The University Animal Care Committee for Animal Research of GNU approved the study protocol (GNU-150116-M0002).

## Supplementary information

Supplementary information.

## Data Availability

The datasets used and/or analyzed during the current study are available from the corresponding author on reasonable request.
