# Supplementary material for: Caloric restriction reverses left ventricular hypertrophy through the regulation of cardiac iron homeostasis in impaired leptin signaling mice
Source: Sci Rep. 2020 Apr 28;10:7176. doi: 10.1038/s41598-020-64201-2 (PMC7188880; doi:10.1038/s41598-020-64201-2)
Supplement: Supplementary file 1 — Supplementary information. [file 41598_2020_64201_MOESM1_ESM.pdf]

Caloric restriction reverses left ventricular hypertrophy through the regulation of cardiac iron homeostasis in impaired leptin signaling mice

Hyeong Seok An[1,2], Jong Youl Lee[1,2], Eun Bee Choi[1,2], Eun Ae Jeong[1,2], Hyun Joo Shin[1,2], Kyung Eun Kim[1,2], Kyung-Ah Park[1,2], Zhen Jin[1,2], Jung Eun Lee[3], Jin Sin Koh[4], Woori Kwak[5], Won-Ho Kim[6], and Gu Seob Roh[1,2]*

Supplementary Table 1. List of qRT-PCR primer

Gene name	Primer sequences
ATF3	Forward 5’ CTCCTGGGTCACTGGTATTTG 3'
	Reverse 5’ CCGATGGCAGAGGTGTTTAT 3'
PGC-1α	Forward 5’ CTAGCCATGGATGGCCTATTT 3'
	Reverse 5’ GTCTCGACACGGAGAGTTAAAG 3'
Tfrc	Forward 5’ TCAAGCCAGATCAGCAATTCTC 3'
	Reverse 5’ AGCCAGTTTCATCTCCACATG 3'
DMT1	Forward 5’ AGGTGAATCGGGCCAATAAG 3'
	Reverse 5’ AAGACGGACACGACAAAGAC 3'
L-Ferritin	Forward 5’ GCCATGGAGAAGAACCTGAA 3'
	Reverse 5’ TTTCCAGGAAGTCACAGAGATG 3'
LCN2	Forward 5’ TCCTCAGGTACAGAGCTACAA 3'
	Reverse 5’ GCTCCTTGGTTCTTCCATACA 3'
24p3r	Forward 5’ TTGCAGGAACTGGAGAATACC 3'
	Reverse 5’ GTTGGTGAAGCCGAGGATAA 3'
hepcidin	Forward 5’ CACCACCTATCTCCATCAACAG 3'
	Reverse 5’ GTTGGTGTCTCTCTTCCTTCTC 3'
Irp1	Forward 5’ TGGGAGTTGTGGGCAAAT 3'
	Reverse 5’ TTCATCAACCGGGAAGAAGG 3'
Irp2	Forward 5’ CGTGTGATTCTGGAGAGCTAAG 3'
	Reverse 5’ CAGGCACTGGTTGCAAATG 3'

Supplementary Table 2. List of primary antibodies

Antibody	Company	Catalog No.	Dilution(s)	Applications	Source
Dysferlin	abcam	ab124684	1:1,000	WB	Rabbit
TGF-β1	Santa Cruz	sc-146	1:1,000	WB	Rabbit
VEGF	Santa Cruz	sc-7269	1:1,000	WB	Mouse
CTGF	abcam	ab6992	1:1,000	WB	Rabbit
MMP9	abcam	ab38898	1:1,000	WB	Rabbit
IL-6	Santa Cruz	sc-57315	1:1,000	WB	Mouse
TLR4	Santa Cruz	sc-16240	1:1,000	WB	Goat
NF-κBp65	Santa Cruz	sc-8008	1:1,000	WB	Mouse
ATF3	Santa Cruz	sc-188	1:1,000	WB	Rabbit
ATF3	abcam	ab58668	1:200	IF	Mouse
NQO1	abcam	ab34173	1:1,000	WB	Rabbit
HO-1	Stressgen	SPA-895	1:1,000	WB	Rabbit
eNOS	BD Bioscienc	BD610296	1:1,000	WB	Mouse
p-eNOS	millipore	07-428-I	1:1,000	WB	Rabbit
LCN2	R&D	AF3508	1:1,000,<br>1:200	WB, IHC	Goat
24p3R	millipore	ABC846	1:1,000	WB	Rabbit
Tfrc	abcam	ab84036	1:1,000	WB	Rabbit
DMT1	Santa Cruz	sc-166884	1:1,000	WB	Mouse
Ferritin	abcam	ab75973	1:2,000	WB	Rabbit
FPN	abcam	ab78066	1:1,000,<br>1:100	WB, IF	Rabbit
Hepcidin	Novusbio	NBP1-59337	1:100	IHC	Rabbit
p84	abcam	ab487	1:3,000	WB	Mouse
α-tubulin	Sigma	T5168	1:5,000	WB	Mouse
IRP1	Santa Cruz	sc-166022	1:1000<br>1:200	WB, IF	Mouse
IRP2	Santa Cruz	sc-33682	1:1000	WB	Mouse

WB, western blot; IF, immunofluorescence; IHC, immunohistochemistry

Supplementary Table 3. Upregulated genes (FDR < 0.001) 40 genes

Gene Symbol	Gene Info	logFC	FDR
**Nnt**	nicotinamide nucleotide transhydrogenase	1.124541	1.69E-21
**Ehd4**	EH-domain containing 4	0.714796	5.34E-07
**Ndrg4**	N-myc downstream regulated gene 4	0.818237	2.74E-08
**Ppargc1a**	peroxisome proliferative activated receptor, gamma, coactivator 1 alpha	1.326248	1.76E-13
**Xirp1**	xin actin-binding repeat containing 1	0.834302	2.10E-09
**Atcayos**	ataxia, cerebellar, Cayman type, opposite strand	1.700281	1.05E-41
**Alas1**	aminolevulinic acid synthase 1	0.975396	1.11E-08
**Rrad**	Ras-related associated with diabetes	0.821386	9.28E-05
**Pik3r1**	phosphatidylinositol 3-kinase, regulatory subunit, polypeptide 1 (p85 alpha)	0.83356	1.35E-05
**Tef**	thyrotroph embryonic factor	0.852512	8.17E-11
**Car14**	carbonic anhydrase 14	0.949252	1.45E-12
**Rbm3**	RNA binding motif protein 3	1.170817	4.53E-15
**Tfdp2**	transcription factor Dp 2	0.963576	2.39E-09
**Ctgf**	connective tissue growth factor	1.153743	1.49E-07
**Usp2**	ubiquitin specific peptidase 2	0.76407	4.07E-08
**Dusp18**	dual specificity phosphatase 18	0.841109	1.28E-06
**Snrpn**	small nuclear ribonucleoprotein N	1.179623	3.91E-24
**Ciapin1**	cytokine induced apoptosis inhibitor 1	0.901474	9.28E-09
**Ifrd1**	interferon-related developmental regulator 1	0.858288	7.38E-08
**Atf3**	activating transcription factor 3	2.377686	3.67E-13
**Fig4**	FIG4 phosphoinositide 5-phosphatase	0.93798	3.64E-14
**Nuak1**	NUAK family, SNF1-like kinase, 1	1.336403	1.39E-27
**Tfrc**	transferrin receptor	1.556791	5.24E-33
**Ccrl2**	chemokine (C-C motif) receptor-like 2	1.6112	8.54E-06
**Ppara**	peroxisome proliferator-activated receptor alpha	0.713286	4.02E-05

**Tmem100**	transmembrane protein 100	0.984801	5.58E-08
**Usp54**	ubiquitin specific peptidase 54	0.800799	1.12E-06
**Sacs**	sacsin	0.888554	8.22E-10
**Klf10**	Kruppel-like factor 10	1.294798	2.03E-10
**Dusp27**	dual specificity phosphatase 27 (putative)	1.027611	1.86E-11
**Zfp568**	zinc finger protein 568	0.981609	3.50E-10
**Zkscan5**	zinc finger with KRAB and SCAN domains 5	0.898488	1.57E-07
**Wdfy1**	WD repeat and FYVE domain containing 1	2.124668	3.72E-39
**Per2**	period circadian clock 2	1.407678	5.46E-15
**Uchl1**	ubiquitin carboxy-terminal hydrolase L1	0.92026	5.07E-06
**Cdo1**	cysteine dioxygenase 1, cytosolic	2.428676	4.21E-15
**Agt**	angiotensinogen (serpin peptidase inhibitor, clade A, member 8)	2.13375	2.70E-09
**Edn3**	endothelin 3	2.421269	7.86E-13
**Snhg11**	small nucleolar RNA host gene 11	6.021534	2.32E-26
**Clec2l**	C-type lectin domain family 2, member L	3.71953	1.04E-09

Supplementary Table 4. Downregulated genes (FDR < 0.001) 26 genes

Gene Symbol	Gene Info	logFC	FDR
**Ano10**	anoctamin 10	-1.39302	8.23E-24
**Pxdn**	peroxidasin	-0.74825	2.52E-07
**Cyr61**	cysteine rich protein 61	-1.53087	2.38E-07
**Pltp**	phospholipid transfer protein	-0.70296	3.13E-05
**Flnb**	filamin, beta	-0.81484	7.34E-06
**Prelp**	proline arginine-rich end leucine-rich repeat	-1.07421	2.03E-14
**Pik3ip1**	phosphoinositide-3-kinase interacting protein 1	-0.71898	1.03E-05
**Myh7b**	myosin, heavy chain 7B, cardiac muscle, beta	-1.0078	2.93E-09
**Sirpa**	signal-regulatory protein alpha	-0.73097	2.80E-05
**Tppp3**	tubulin polymerization-promoting protein family member 3	-1.01835	1.12E-06
**Abcc5**	ATP-binding cassette, sub-family C (CFTR/MRP), member 5	-0.71111	8.17E-05
**Abca9**	ATP-binding cassette, sub-family A (ABC1), member 9	-0.79933	6.58E-05
**Aqp7**	aquaporin 7	-0.89514	1.16E-06
**Sp100**	nuclear antigen Sp100	-1.01242	6.62E-11
**Pdia4**	protein disulfide isomerase associated 4	-0.8013	4.10E-06
**Adgre1**	adhesion G protein-coupled receptor E1	-1.31361	1.04E-05
**Ifi203**	interferon activated gene 203	-1.42847	4.26E-14
**Hmcn2**	hemicentin 2	-1.69932	9.31E-11
**Mndal**	myeloid nuclear differentiation antigen like	-1.16996	2.28E-09
**Hdac9**	histone deacetylase 9	-1.12443	2.78E-10
**Uba7**	ubiquitin-like modifier activating enzyme 7	-1.26078	3.93E-09
**Akr1c14**	aldo-keto reductase family 1, member C14	-1.43389	9.43E-11
**Gbp7**	guanylate binding protein 7	-1.2885	6.76E-06
**Psmb9**	proteasome (prosome, macropain) subunit, beta type 9 (large multifunctional peptidase 2)	-1.39063	4.24E-08

**Slc16a7**	solute carrier family 16 (monocarboxylic acid transporters), member 7	-1.58091	8.60E-07
**Apol10b**	apolipoprotein L 10B	-1.69981	4.36E-05

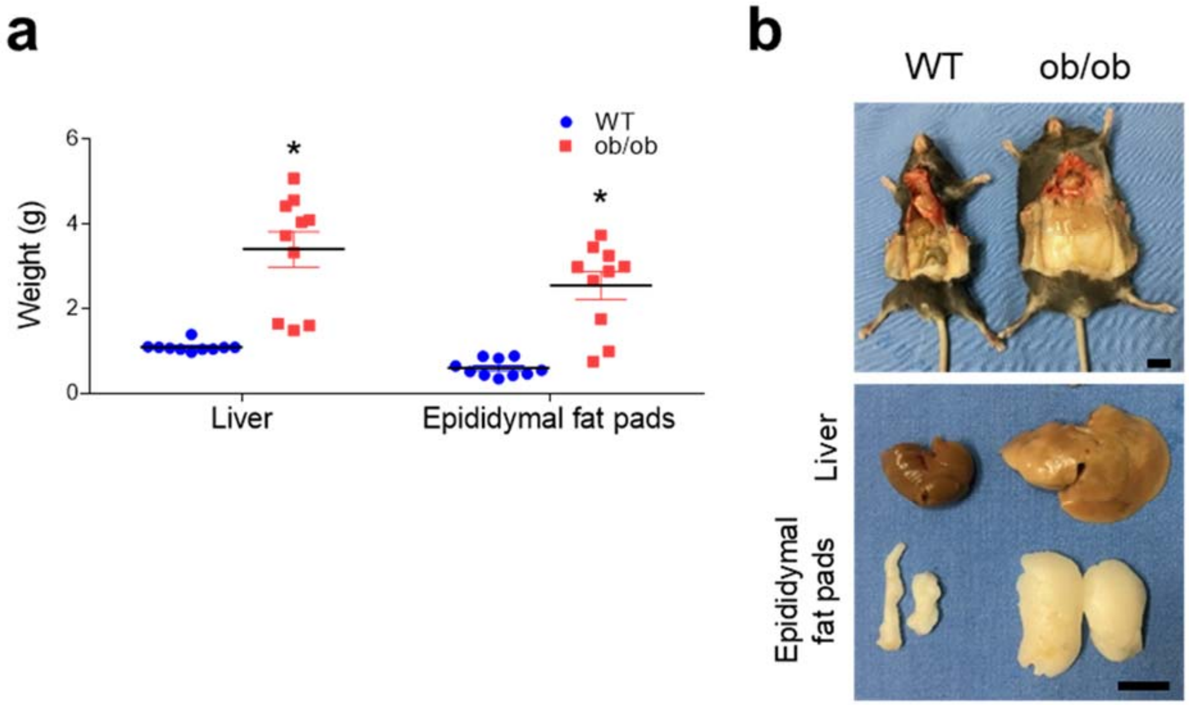

Supplementary Figure 1. Physiological characteristics of WT and ob/ob mice. (**a**) Weight of liver and epididymal fat pads in WT and ob/ob mice. (**b**) Gross morphology of intraabdominal organs, liver, and epididymal fat pads, demonstrating fatty liver and dramatically enlarged epididymal fat pad in ob/ob relative to WT mice. Data (n = 10 mice per group) are shown as mean ± SEM. *P < 0.05 vs. WT. Statistical analysis was carried out by a Student's t-test.

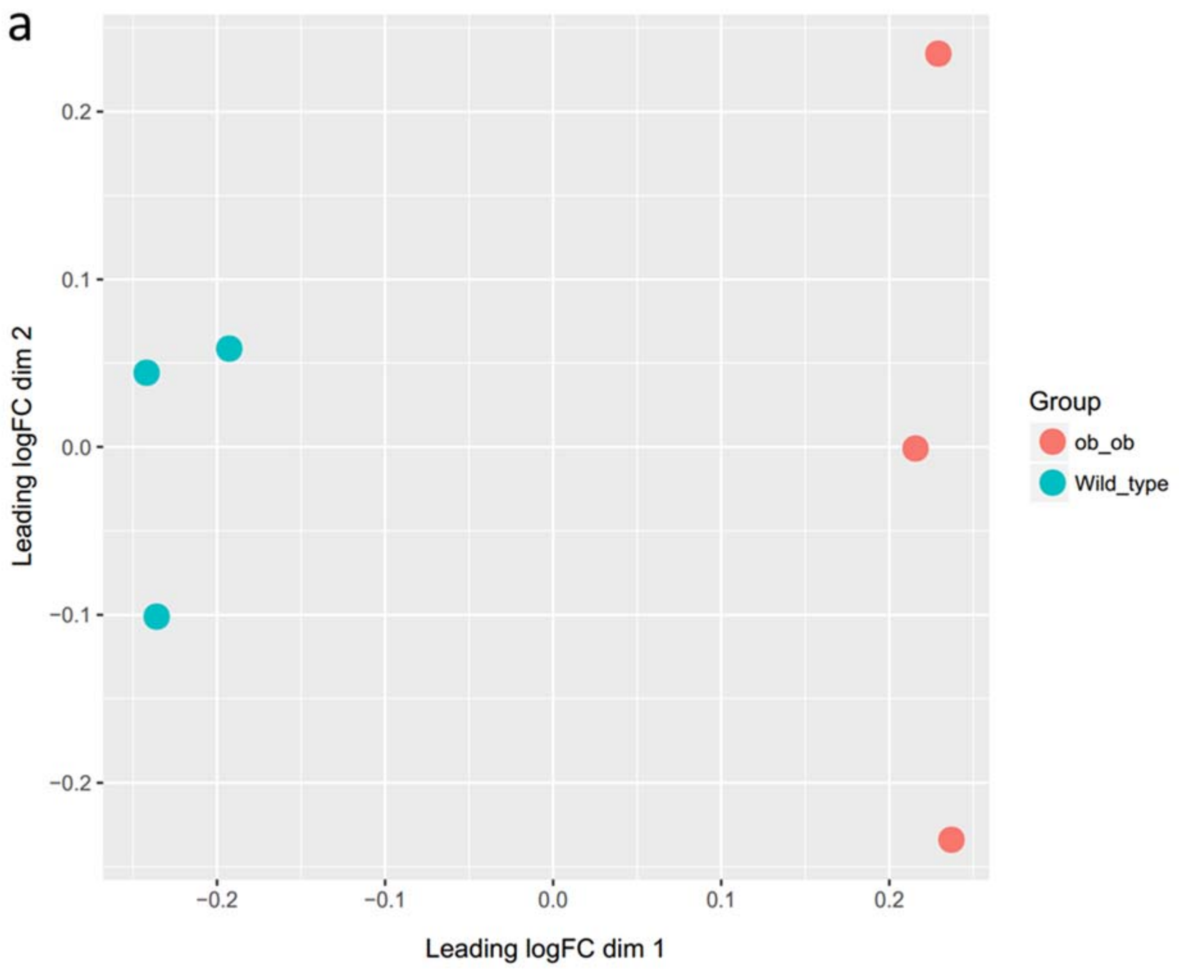

Supplementary Figure 2a. Gene expression profile of RNA-seq analysis in the hearts of wild-type (WT) and obese (ob/ob) mice. Principal component analysis plot of the samples (n = 3 mice per group).

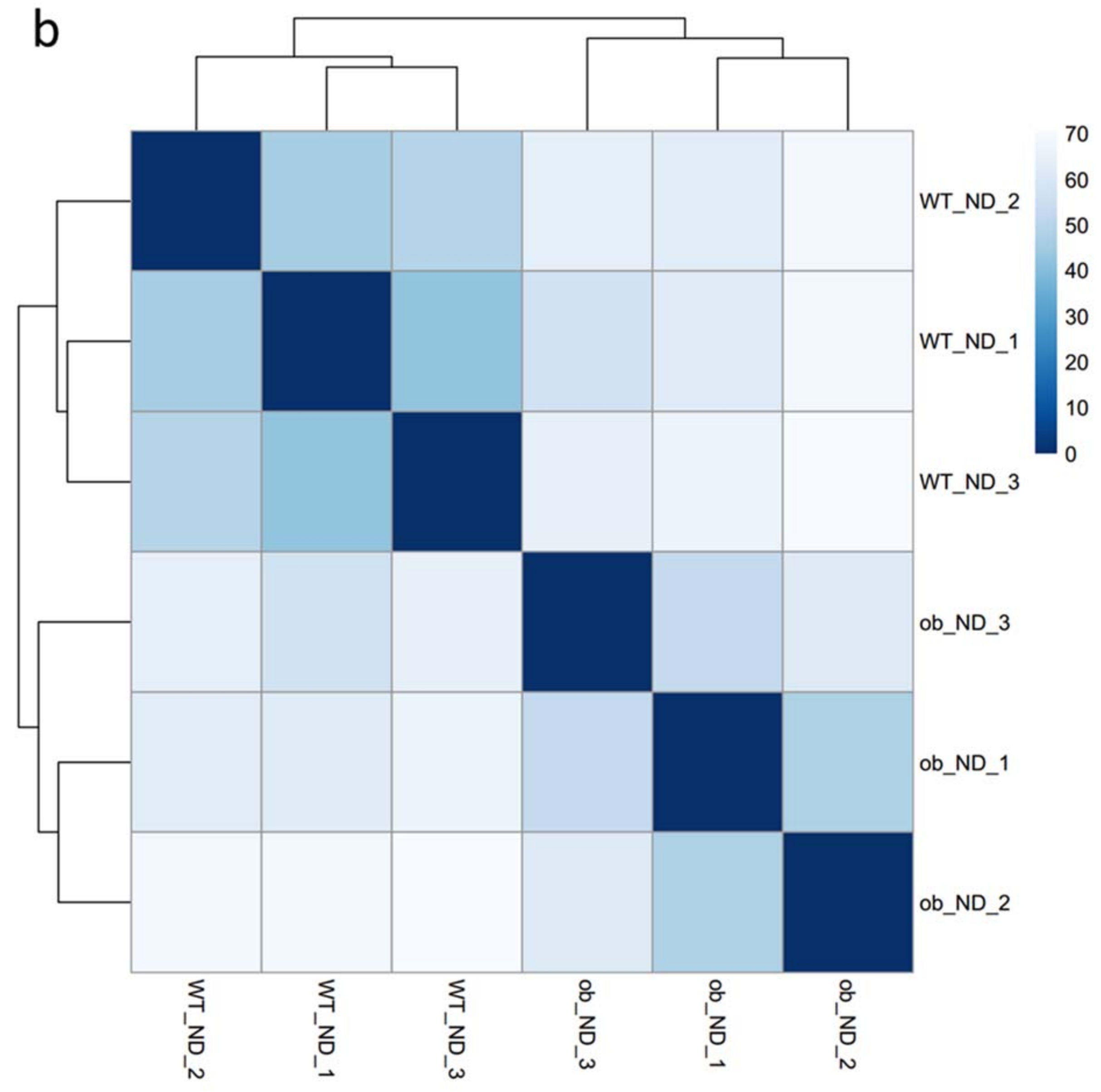

Supplementary Figure 2b. Gene expression profile of RNA-seq analysis in the hearts of wild-type (WT) and obese (ob/ob) mice. Heatmap of sample-to-sample distances (n = 3 mice per group).

C

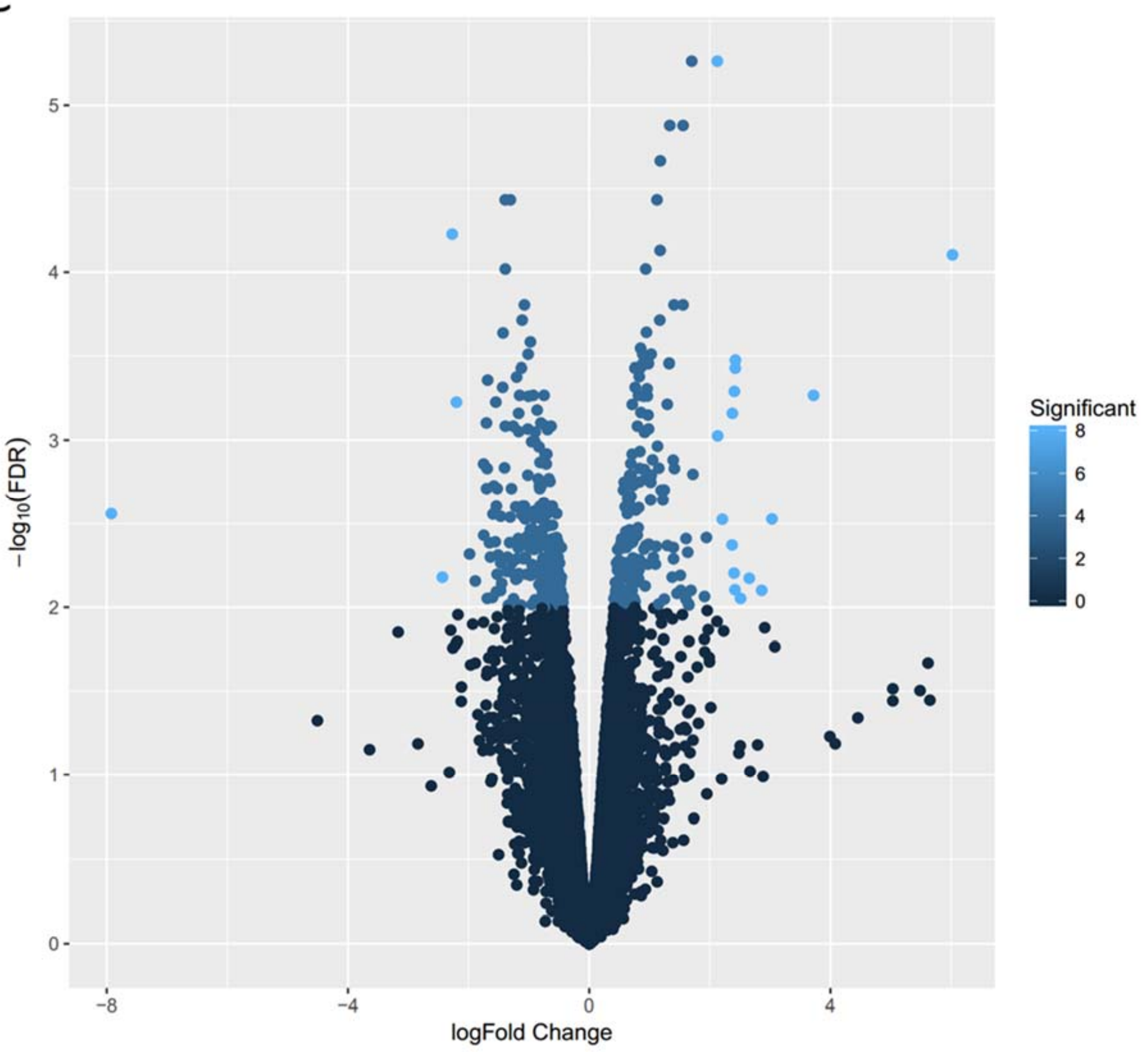

Supplementary Figure 2c. Gene expression profile of RNA-seq analysis in the hearts of wild-type (WT) and obese (ob/ob) mice. Volcano plot for differentially expressed gene analysis using EdgeR (n = 3 mice per group).

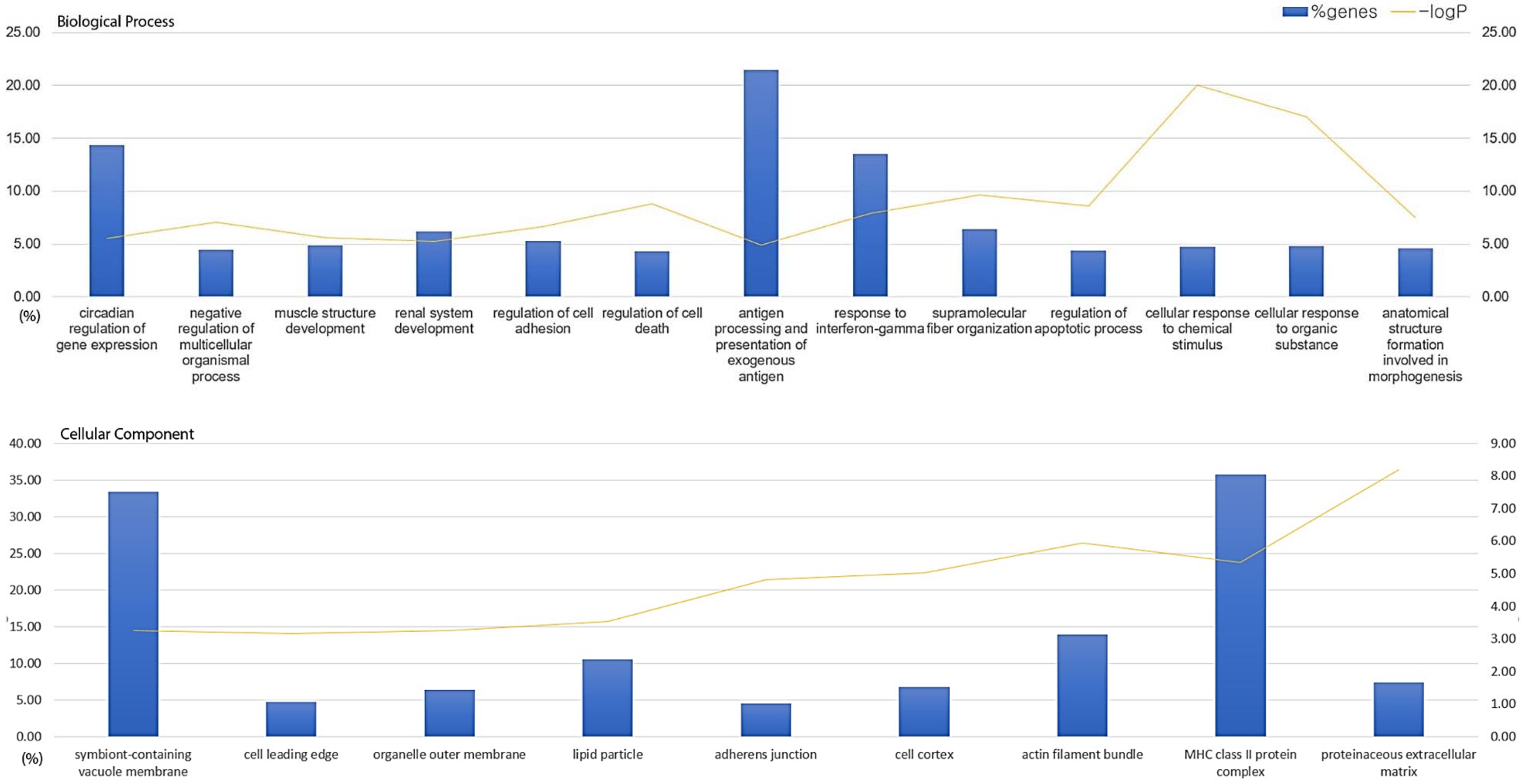
Biological Process
%genes
−logP
25.00
20.00
15.00
10.00
5.00
0.00
(%)
circadian regulation of gene expression
negative regulation of multicellular organismal process
muscle structure development
renal system development
regulation of cell adhesion
regulation of cell death
antigen processing and presentation of exogenous antigen
response to interferon-gamma
supramolecular fiber organization
regulation of apoptotic process
cellular response to chemical stimulus
cellular response to organic substance
anatomical structure formation involved in morphogenesis
Cellular Component
40.00
35.00
30.00
25.00
20.00
15.00
10.00
5.00
0.00
(%)
9.00
8.00
7.00
6.00
5.00
4.00
3.00
2.00
1.00
0.00
symbiont-containing vacuole membrane
cell leading edge
organelle outer membrane
lipid particle
adherens junction
cell cortex
actin filament bundle
MHC class II protein complex
proteinaceous extracellular matrix

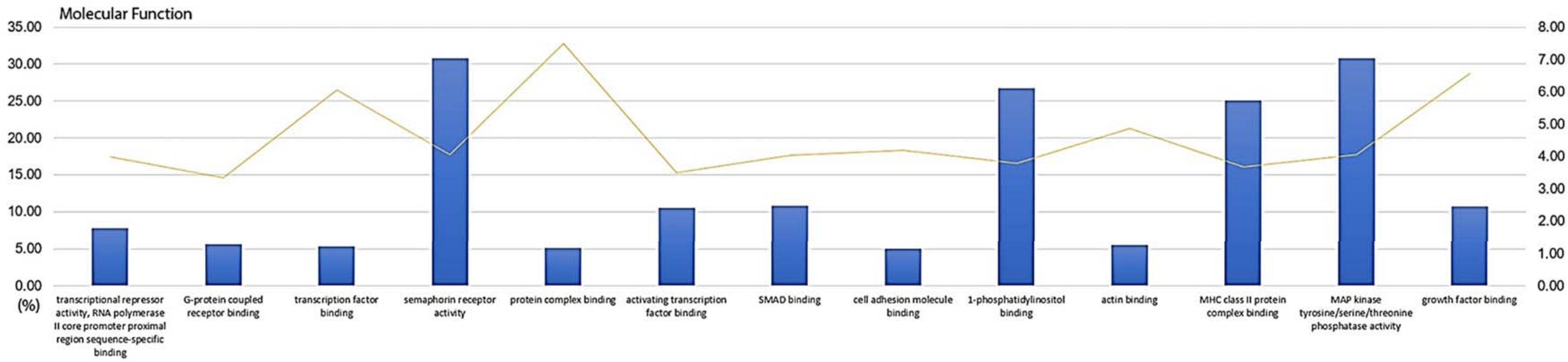

Supplementary Figure 3. Enriched GO terms (biological process, cellular component and molecular function) using ClueGO pathway enrichment analysis in the hearts of wild-type (WT) and obese (ob/ob) mice. Only representative GO terms that include more than two sibling terms are displayed. Blue bars indicate the proportion of associated genes, and yellow lines indicate -log (P-value) of the enrichment analysis.

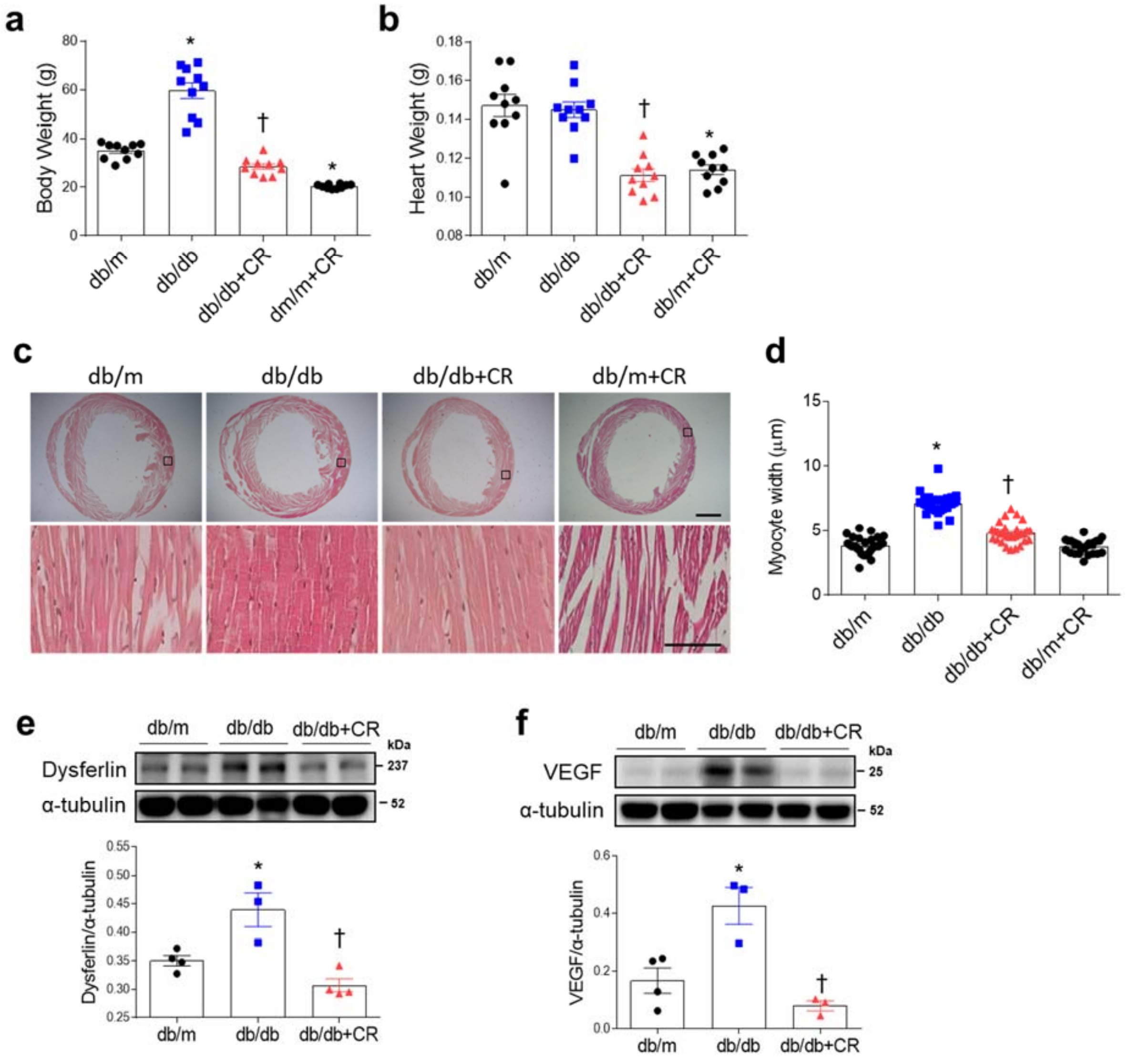

Supplementary Figure 4. Effect of CR on cardiac hypertrophy and fibrosis in db/db mice. (**a**) Body weight and (**b**) heart weight in db/m, diabetic db/db, diabetic calorie-restricted (db/db+CR), and calorie-restricted db/m (db/m+CR) mice. (n = 10 per group). (**c**) Representative images of H&E staining in heart sections showing cardiomyocytes (scale bar = 1,000 μm [above], 100 μm [below]). (**d**) Cardiomyocyte width, as measured from histological sections. Western blots and quantitative analysis showing expression of cardiac fibrotic markers of dysferlin (**e**) and VEGF (**f**) (n = 4–5 per group). Cropped blots are displayed here and full-length blots are included in the Supplementary Information. Data are shown as mean

± SEM. *$P < 0.05$ vs. db/m mice. †$P < 0.05$ vs. db/db mice. Statistical analysis was carried out by one-way ANOVA followed by Bonferroni post hoc analysis.

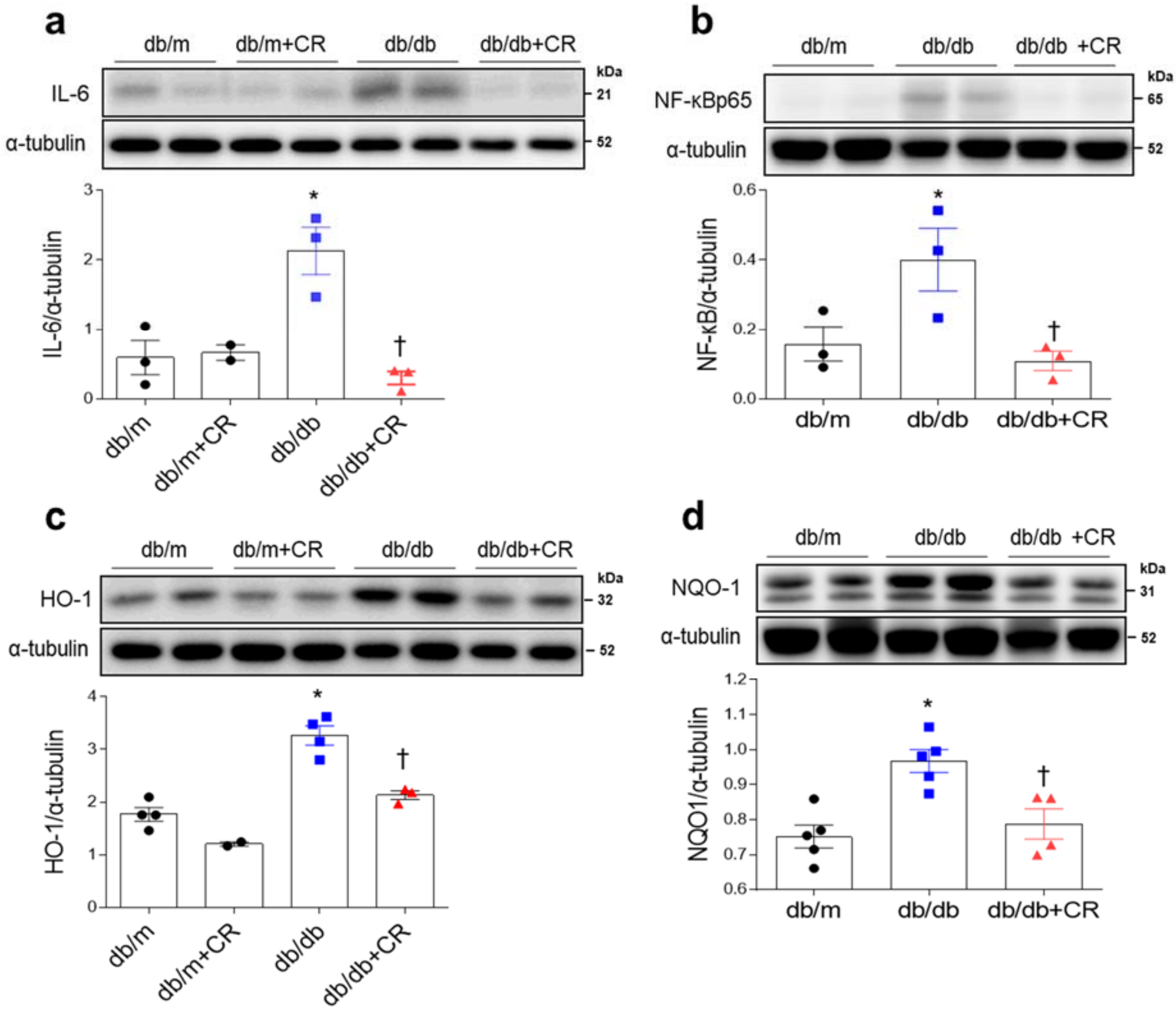

Supplementary Figure 5. Effect of CR on cardiac inflammation and oxidative stress in db/db mice. Western blots and quantitative analysis showing expression of cardiac IL-6 (**a**), NF-κBp65 (**b**), HO-1 (**c**), and NQO-1 (**d**) in db/m, diabetic db/db, diabetic calorie-restricted (db/db+CR), and calorie-restricted db/m (db/m+CR) mice (n = 4–5 per group). Cropped blots are displayed here and full-length blots are included in the Supplementary Information. Data are shown as mean ± SEM. *$P < 0.05$ vs. db/m mice. $^{\dagger}P < 0.05$ vs. db/db mice. Statistical analysis was carried out by one-way ANOVA followed by Bonferroni post hoc analysis.

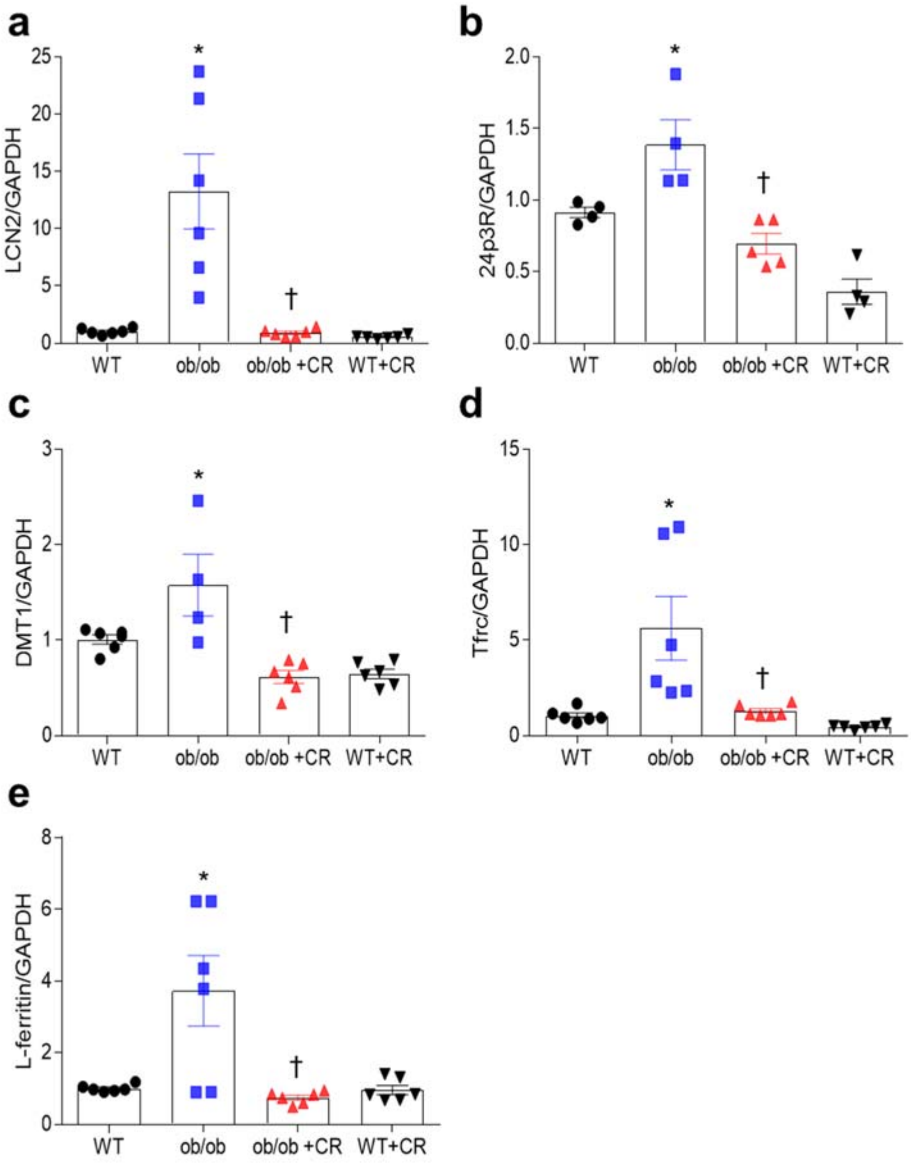

Supplementary Figure 6. Effect of CR on iron-related gene mRNA expression in the hearts of ob/ob mice. Quantitative RT-PCR analysis of LCN2 (**a**), 24p3R (**b**), DMT1 (**c**), Tfrc (**d**), and ferritin (**e**) expression in wild-type (WT), obese (ob/ob), obese calorie-restricted (ob/ob+CR), and calorie restricted WT (WT+CR) mice. Data (n = 4–6 per group) are shown as mean ± SEM. *P < 0.05 vs. WT mice. †P < 0.05 vs. ob/ob mice. Statistical analysis was carried out by one-way ANOVA followed by Bonferroni post hoc analysis.

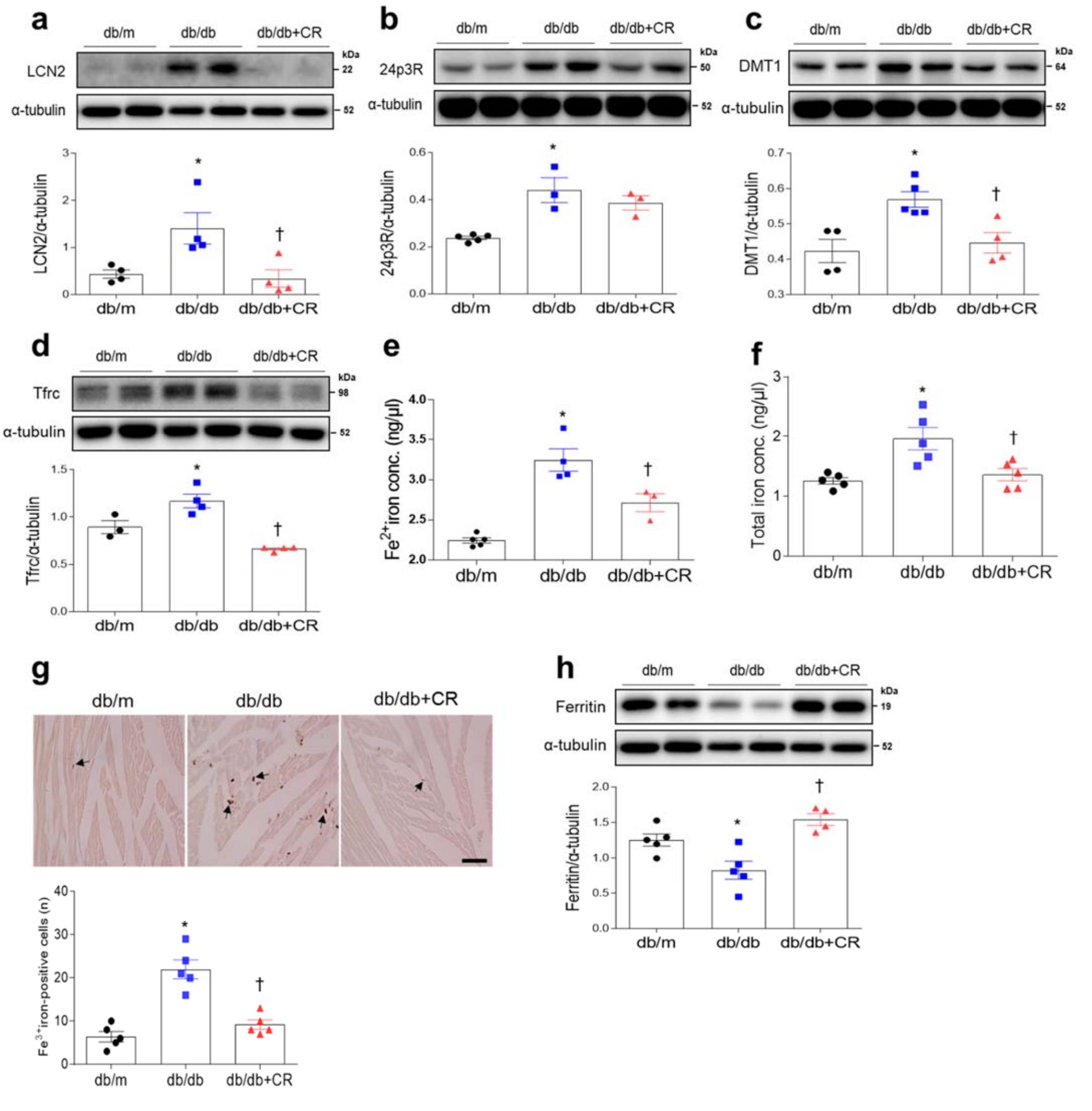

Supplementary Figure 7. Effect of CR on iron transporter-related proteins in the hearts of db/db mice. Western blots and quantitative analysis showing expression of cardiac LCN2 (**a**), 24p3R (**b**), DMT1 (**c**), and Tfrc (**d**) in db/m, diabetic db/db, and diabetic calorie-restricted (db/db+CR) mice (n = 3–5 per group). Iron assay and quantitative analysis showing cardiac

ferrous Fe^{2+} (**e**) and total iron (**f**) levels (n = 3–6 per group). (**g**) Representative images of DAB-enhanced Perls' staining for ferric iron (Fe^{3+}). The numbers of Fe^{3+}iron-positvie cells were measured in Perl's staining for heart sections (scale bar = 50 μm). (**h**) Western blots and quantitative analysis showing cardiac ferritin expression mice (n = 4–5 per group). Cropped blots are displayed here and full-length blots are included in the Supplementary Information. Data are shown as mean ± SEM. *$P < 0.05$ vs. db/m mice. $^{\dagger}P < 0.05$ vs. db/db mice. Statistical analysis was carried out by one-way ANOVA followed by Bonferroni post hoc analysis.

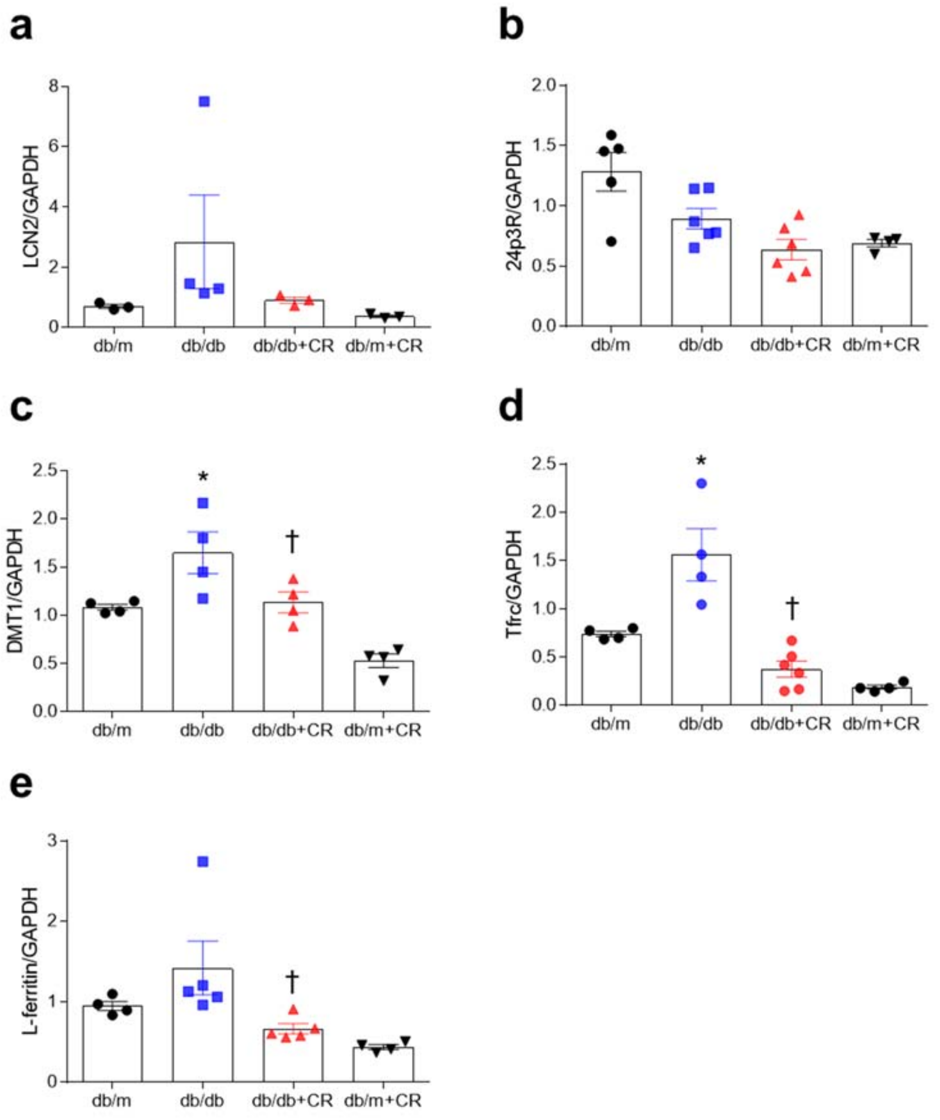

Supplementary Figure 8. Effect of CR on iron-related gene mRNA expression in the hearts of db/db mice. Quantitative RT-PCR analysis of cardiac LCN2 (**a**), 24p3R (**b**), DMT1 (**c**), Tfrc (**d**), and ferritin (**e**) in db/m, diabetic db/db, diabetic calorie-restricted (db/db+CR) mice and calorie-restricted db/m (db/m+CR) mice (n = 3–6 per group). Data are shown as mean ± SEM. *$P < 0.05$ db/m mice. $^{\dagger}P < 0.05$ vs. db/db mice. Statistical analysis was carried out by one-way ANOVA followed by Bonferroni post hoc analysis.

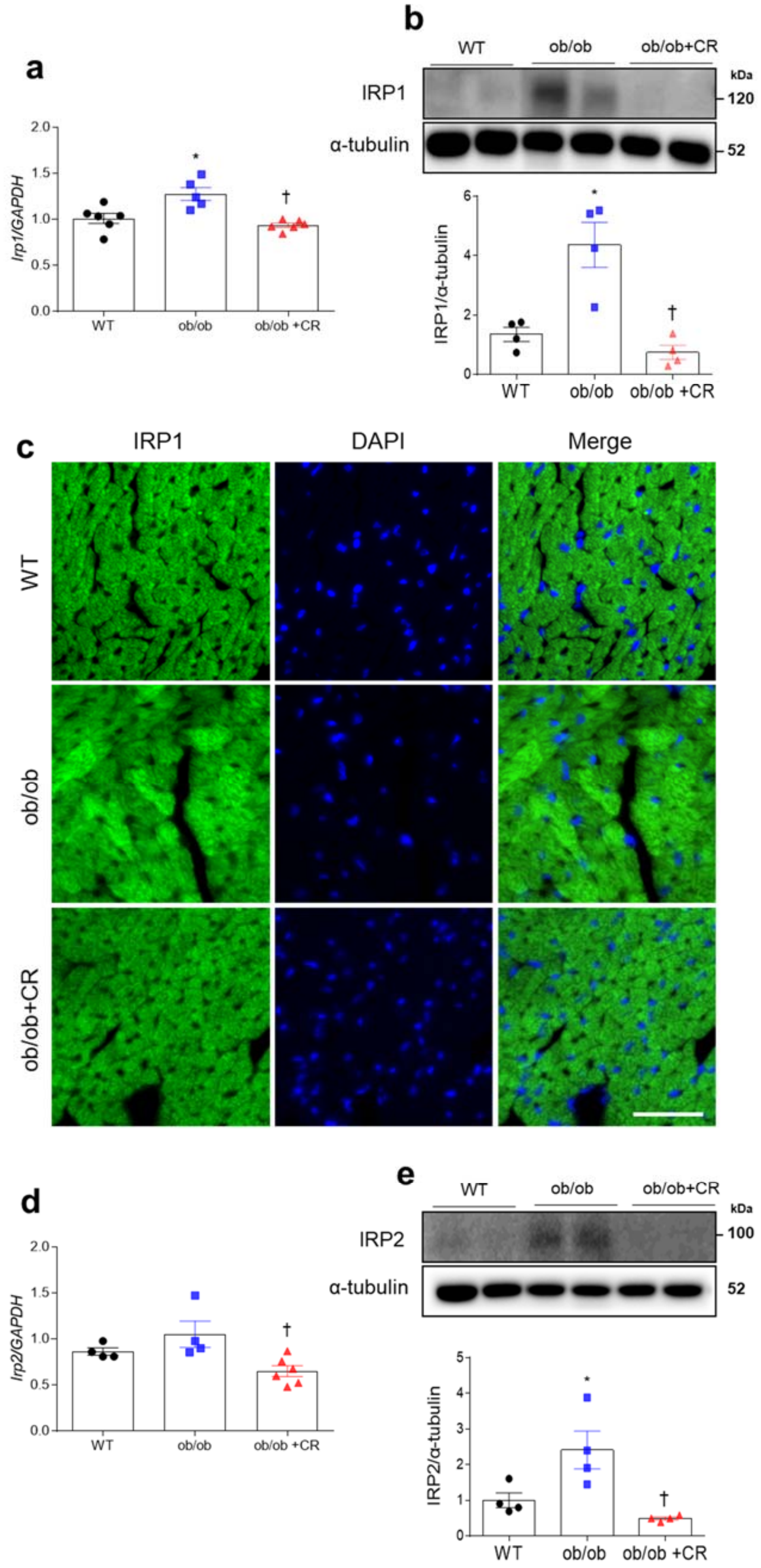

a
Irp1/GAPDH
2.0
1.5
1.0
0.5
0.0
WT
ob/ob
ob/ob +CR
b
WT
ob/ob
ob/ob+CR
IRP1
α-tubulin
kDa
120
52
IRP1/α-tubulin
6
4
2
0
WT
ob/ob
ob/ob +CR
c
IRP1
DAPI
Merge
WT
ob/ob
ob/ob+CR
d
Irp2/GAPDH
2.0
1.5
1.0
0.5
0.0
WT
ob/ob
ob/ob +CR
e
WT
ob/ob
ob/ob+CR
IRP2
α-tubulin
kDa
100
52
IRP2/α-tubulin
5
4
3
2
1
0
WT
ob/ob
ob/ob +CR

Supplementary Figure 9. Effect of CR on cardiac IRP1 and IRP2 expressions in ob/ob mice. (**a**) Quantitative RT-PCR analysis of cardiac *Irp1* mRNA (n = 5–6 per group). (**b**) Western blots and quantitative analysis showing cardiac IRP1 in wild type (WT), ob/ob, and calorie-restricted (ob/ob+CR) mice. (**c**) Representative images of immunofluorescence staining for IRP1 from heart sections. Nuclei were stained with DAPI. Scale bar = 25 μm. (**d**) Quantitative RT-PCR analysis of cardiac *Irp2* mRNA (n = 4–6 per group). (**e**) Western blots and quantitative analysis showing cardiac IRP2. Cropped blots are displayed here and full-length blots are included in the Supplementary Information. Data (n = 4 per group) are shown as mean ± SEM. *$P < 0.05$ vs. WT mice. †$P < 0.05$ vs. ob/ob mice. Statistical analysis was carried out by one-way ANOVA followed by Bonferroni post hoc analysis.

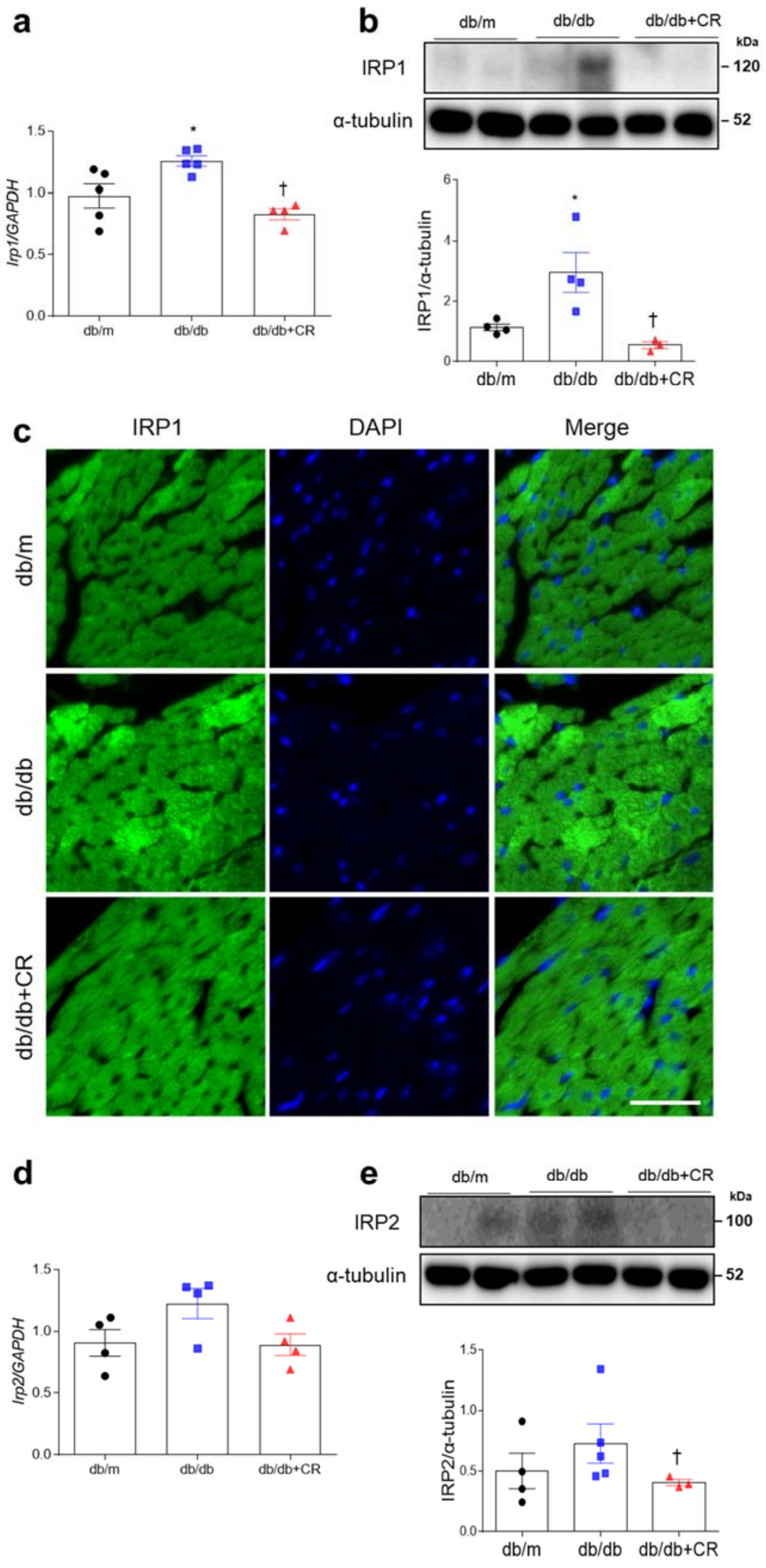
a
b
db/m
db/db
db/db+CR
kDa
IRP1
120
α-tubulin
52
Irp1/GAPDH
IRP1/α-tubulin
c
IRP1
DAPI
Merge
d
e
IRP2
100
Irp2/GAPDH
IRP2/α-tubulin

Supplementary Figure 10. Effect of CR on cardiac IRP1 and IRP2 expressions in db/db mice. (**a**) Quantitative RT-PCR analysis of cardiac *Irp1* mRNA (n = 4–5 per group). (**b**) Western blots and quantitative analysis showing cardiac IRP1 in in db/m, diabetic db/db, and diabetic calorie-restricted (db/db+CR) mice. (**c**) Representative images of immunofluorescence staining for IRP1 from heart sections. Nuclei were stained with DAPI. Scale bar = 25 μm. (**d**) Quantitative RT-PCR analysis of cardiac *Irp2* mRNA (n = 4 per group). (**e**) Western blots and quantitative analysis showing cardiac IRP2. Cropped blots are displayed here and full-length blots are included in the Supplementary Information. Data (n = 4 per group) are shown as mean ± SEM. *P < 0.05 vs. db/m mice. †P < 0.05 vs. db/db mice. Statistical analysis was carried out by one-way ANOVA followed by Bonferroni post hoc analysis.

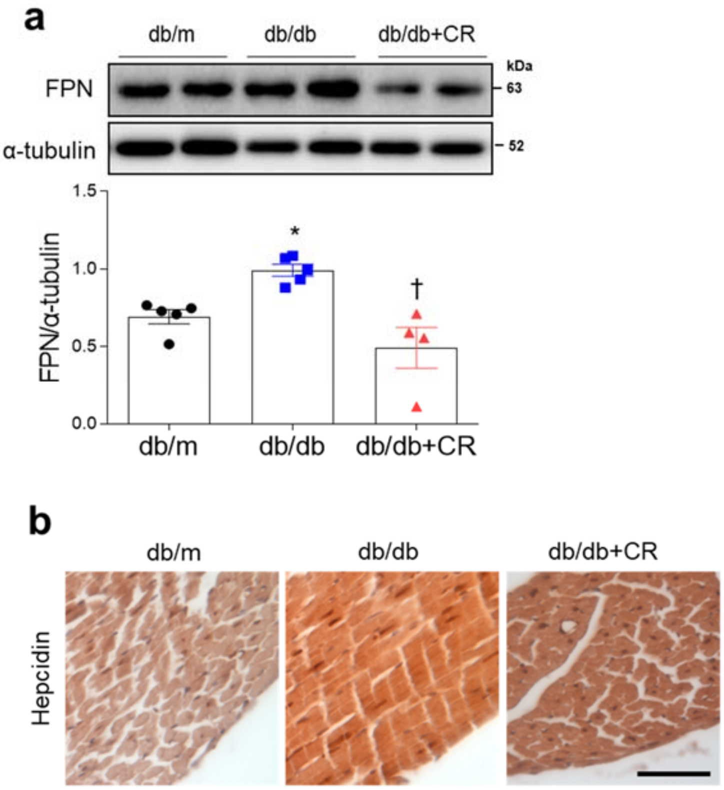

Supplementary Figure 11. Effect of CR on cardiac ferroportin and hepcidin expressions in db/db mice. (**a**) Western blots and quantitative analysis showing cardiac ferroportin (FPN) in db/m, diabetic db/db, and diabetic calorie-restricted (db/db+CR) mice. Cropped blots are displayed here and full-length blots are included in the Supplementary Information. (**b**) Representative images of immunohistochemistry for hepcidin in heart sections (Scale bar = 50 μm). Data (n = 4–5 per group) are shown as mean ± SEM. *P < 0.05 vs. db/m mice. †P < 0.05 vs. db/db mice. Statistical analysis was carried out by one-way ANOVA followed by Bonferroni post hoc analysis.

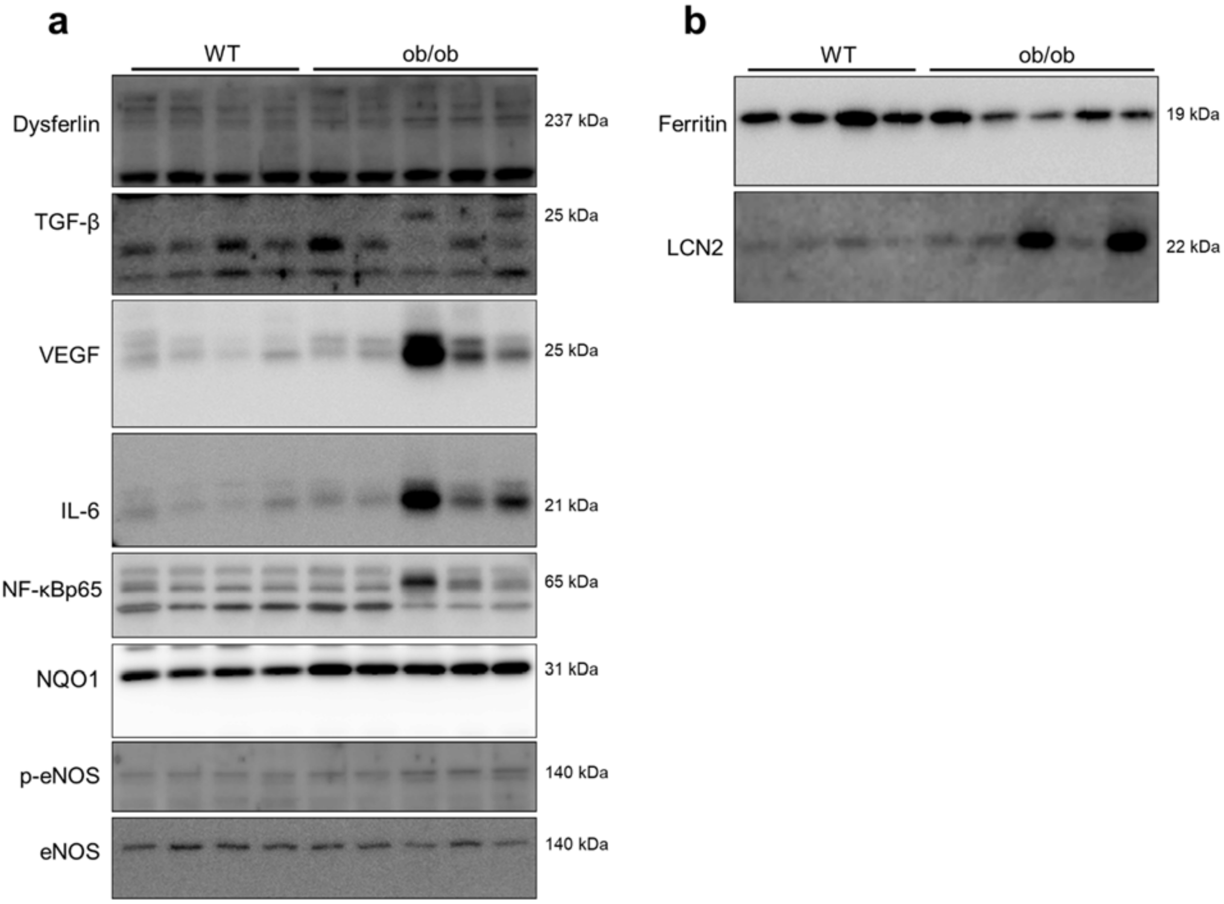

Supplementary Figure 12. (a) Full-length blots of figure 1h-n. **(b)** Full length blots of figure 2g-h.

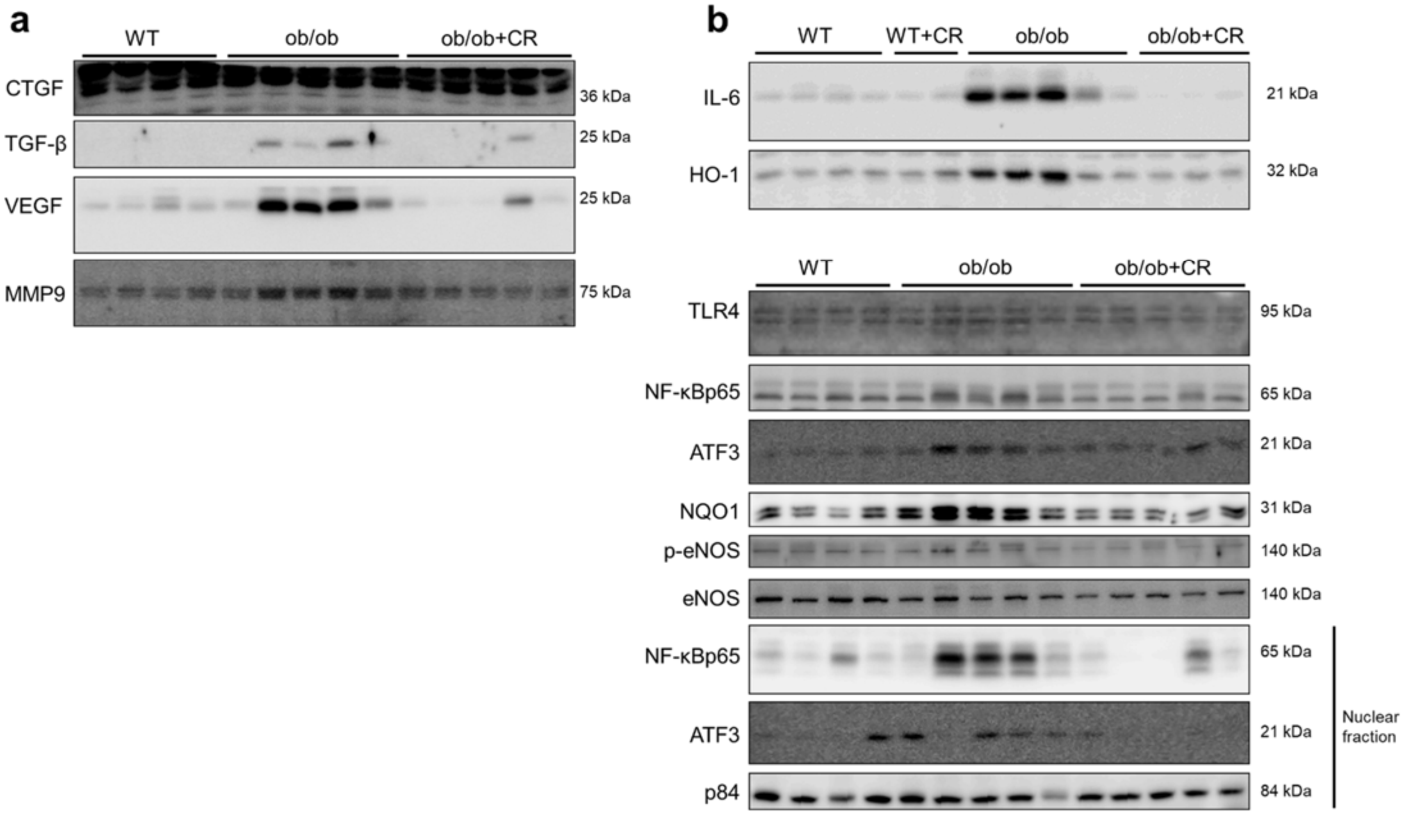

Supplementary Figure 13. (a) Full-length blots of figure 3e-h. **(b)** Full length blots of figure 4a-i.

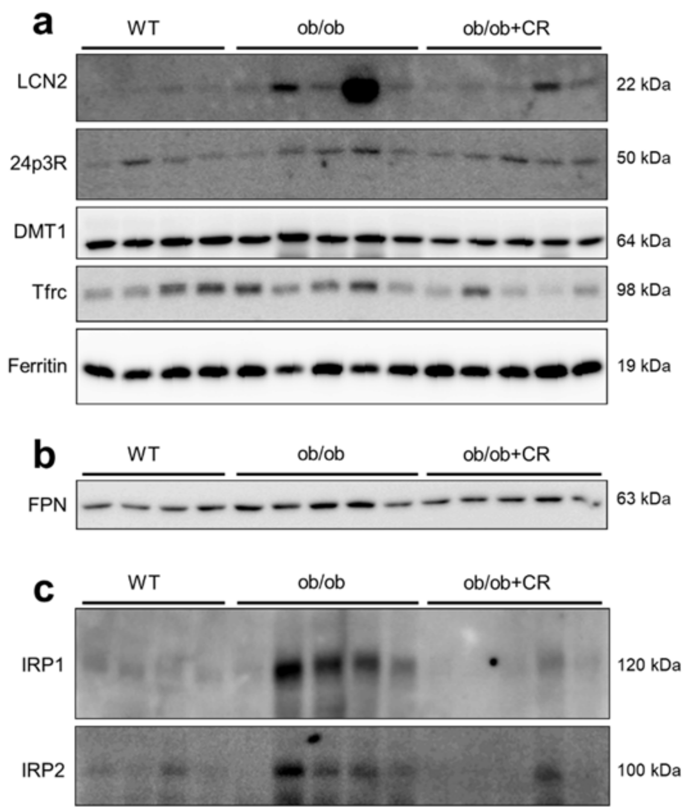

Supplementary Figure 14. (a) Full-length blots of figure 5a-h. **(b)** Full length blots of figure 6a. **(c)** Full length blots of figure S9b and e.

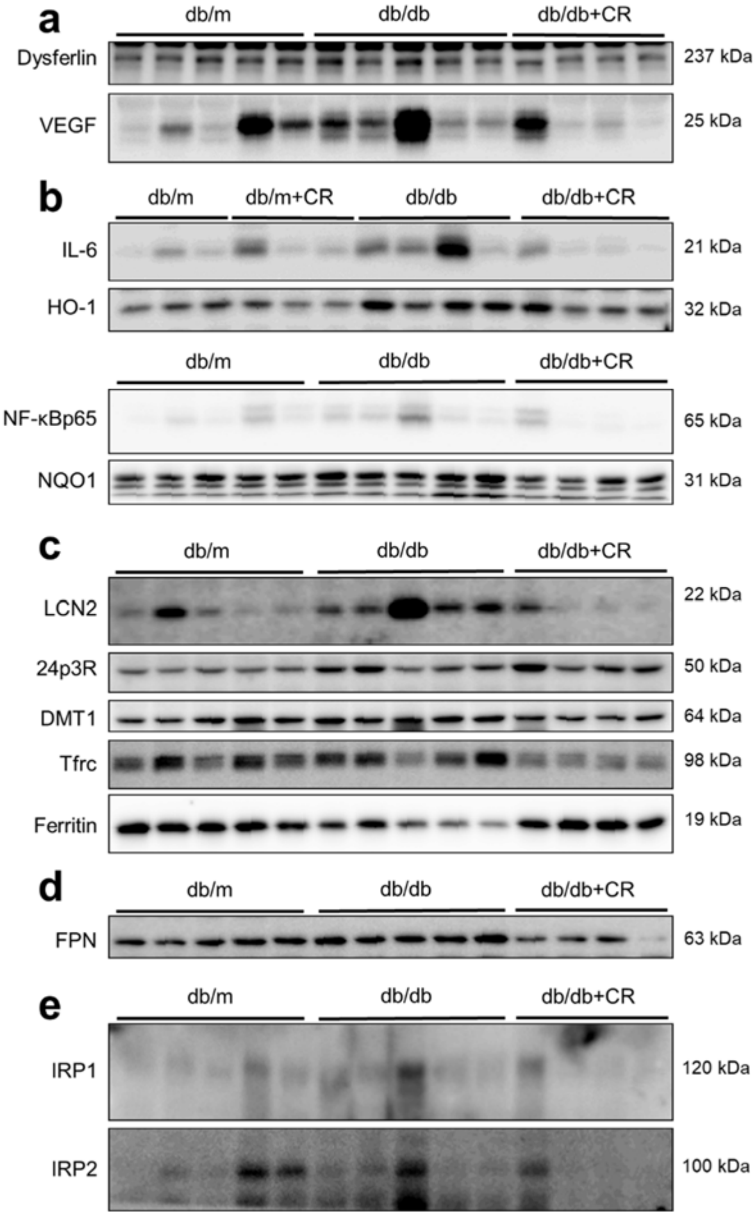

Supplementary Figure 15. (a) Full-length blots of figure S5e-f. **(b)** Full length blots of figure S6a-d. **(c)** Full length blots of figure S7a-g. **(d)** Full length blots of figure S9a. **(e)** Full length blots of figure S10b and e.
